# Multipolar model and Hirshfeld atom refinement of tetra­aqua­bis­(hydrogenmaleato)iron(II)

**DOI:** 10.1107/S2052520625003403

**Published:** 2025-05-09

**Authors:** Hellen Ferreira Guimarães, Bernardo Lages Rodrigues

**Affiliations:** a Department of Chemistry Federal University of Minas Gerais Avenida Antonio Carlos 6627 Belo Horizonte Minas Gerais31310-390 Brazil; Warsaw University, Poland

**Keywords:** aspherical refinements, topological analysis, hydrogen bonds, multipole model, Hirshfeld atom refinement

## Abstract

A high-resolution charge density study using the Hansen–Coppens multipolar model was performed on tetra­aqua­bis­(hydrogenmaleato)iron(II). The experimental electron density was subjected to Bader’s topological analysis. Hirshfeld atom refinement and topological analysis of the molecular wavefunction were also conducted. A comparison of the properties obtained under different resolution and acquisition conditions are presented. The performance of these models is evaluated in terms of their ability to achieve bond lengths close to those from neutron diffraction, provide accurate anisotropic displacement parameters and model electron densities precisely, and to determine atomic charges under different experimental and modeling conditions. The structure presents a short intramolecular hydrogen bond, which is found to have a distinct character compared to other interactions, as the hydrogen interacts covalently with two oxygen atoms. Different models were evaluated, each outperforming the others in specific aspects. Overall, the analysis of these models provide deeper insights into electron density distribution and the nature of the interactions present in the structure.

## Abstract 2

Multipolar model and Hirshfeld atom refinement are conducted on tetra­aqua­bis(hydrogenmaleato)iron(II) based on a high-resolution X-ray diffraction experiment. Topological analysis is performed on both models. The study evaluates these models by comparing their effectiveness in determining bond lengths, anisotropic displacement parameters, electron densities, and atomic charges.

## 1. Introduction

Understanding chemical bonds and interactions is crucial for interpreting and predicting reactions and modeling properties of interest since the characteristics of compounds directly result from the electron arrangement of their atoms. For coordination compounds and intra- and intermolecular interactions in particular, the nature of the contacts can include ionic, covalent, and mixed characters, which is challenging for traditional models to interpret electron distribution. Advancements in experimental techniques and refinement methods have greatly enhanced the comprehension of the interactions in crystalline materials, providing important information about the chemical bonds and crystal packing.

The independent atom model (IAM) refinement approach has traditionally been effective for the positioning of heavy atoms, which have minimal contribution from valence electrons. However, this model often results in inaccurate bond lengths involving lighter atoms, particularly for hydrogen, which does not have inner electron shells, and its only electron is involved in a chemical bond. Consequently, applying this model results in an apparent shortening of *X*–H, where *X* is another atom bonded to the hydrogen atom (Coppens, 1997 ▸). On the other hand, the neutron diffraction experiment is well known for its ability to provide precise atomic positions and displacement parameters, including for hydrogen atoms, since neutrons primarily interact with atomic nuclei.

To accurately describe the electron density and obtain atomic positions comparable to those from neutron diffraction, it is essential to treat atoms aspherical entities (Woińska *et al.*, 2016 ▸; Dittrich *et al.*, 2017 ▸; Hoser *et al.*, 2009 ▸). In this regard, the multipolar model (MM) of Hansen & Coppens (1978 ▸) is widely used. In this model, the atomic electron density (ρ_a_) is described as a combination of core spherical density, spherical valence density, and aspherical valence deformation density [equations (1) and (2)]. The latter uses a multipole expansion based on spherical harmonics whose coefficients are refined against experimental data. The second and third terms have refinable parameters (κ and κ′, respectively) that account for the contraction or expansion of the respective valence density:
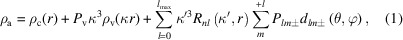
where the radial functions are
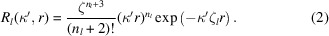
Although MM usually enables an accurate description of electron density for certain species, it requires rigorous experimental conditions, such as high-resolution and low-temperature measurements, and small-size, high-quality single crystals, besides being highly user dependent (Koritsanszky & Coppens, 2001 ▸).

Advances in quantum crystallography—a field defined by the mutual enhancement of crystallographic data and quantum mechanical calculations (Massa, Huang & Karle, 1995 ▸; Grabowsky *et al.*, 2017 ▸; Genoni *et al.*, 2018 ▸)—have enabled the application of alternative aspherical atom models. One of those approaches is Hirshfeld atom refinement (HAR) (Jayatilaka & Dittrich, 2008 ▸; Capelli *et al.*, 2014 ▸), which combines X-ray diffraction experimental data with *ab initio* calculation. HAR employs molecular wavefunction computation. This wavefunction is partitioned using the Hirshfeld stockholder partitioning scheme (Hirshfeld, 1977 ▸). The resulting aspherical atomic electron densities are used to compute aspherical structure factors, which are then applied in the refinement of structural parameters against experimental data. This procedure is done iteratively until the convergency is achieved. Equations (3) and (4) show the Hirshfeld partitioning scheme, where ρ_A_(**r**) is the electron density associated with atom A, *w*_A_(**r**) is a weighting function that determines the fraction of the molecular electron density ρ_mol_(**r**) assigned to atom A. The weighting function *w*_A_(**r**) is defined as the ratio of the spherically averaged electron density of atom A, 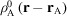, at position **r**_A_, to the sum of the spherically averaged electron densities of all atoms in the molecule, 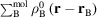:
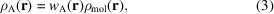
where
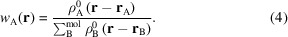
Recent developments, such as the *NoSpherA2* (Kleemiss *et al.*, 2021 ▸) module in *Olex2* software (Dolomanov *et al.*, 2009 ▸) have made the implementation of HAR more accessible. The new capabilities allow, for instance, the application of quantum chemistry software packages such as *ORCA5.0* (Neese, 2022 ▸), restraints and constraints from *olex2.refine*, and disorder treatment (Kleemiss *et al.*, 2024 ▸; Hatcher *et al.*, 2023 ▸; Jha *et al.*, 2023 ▸). These advancements, along with the ability of HAR to provide *X*–H distances and displacement parameters in good agreement with those from neutron diffraction, even for non-high-resolution X-ray diffraction experiments, extend its applicability to a broader range of systems and common experimental conditions (Chęcińska *et al.*, 2013 ▸; Woińska *et al.*, 2014 ▸).

Other alternatives to IAM have already been proposed. For instance, the transferable aspherical atom model (TAAM) (Jha *et al.*, 2020 ▸; Volkov *et al.*, 2007 ▸; Dominiak *et al.*, 2007 ▸; Bojarowski *et al.*, 2022 ▸), which is based on transferring the multipolar parameters of the pseudo atoms from one molecule to pseudo atoms in other molecules with similar chemical environments (Brock *et al.*, 1991 ▸). However, fragments containing transition metals are not yet included in existing compiled databases (Sanjuan-Szklarz *et al.*, 2020 ▸). To overcome this limitation, hybrid methods such as IAM–TAAM, HAR–TAAM, and HAR joined with extremely localized molecular orbitals (ELMOs) (Meyer *et al.*, 2016 ▸; Meyer & Genoni, 2018 ▸)—HAR–ELMO (Malaspina *et al.*, 2019 ▸; Malaspina *et al.* 2021*a* ▸)—have emerged. Additionally, models such as X-ray constrained wavefunction (Jayatilaka & Grimwood, 2001 ▸) and X-ray wavefunction refinement (Grabowsky *et al.*, 2012 ▸) have been demonstrated to produce highly accurate electron density models by incorporating quantum-mechanical wavefunctions directly into the refinement process.

Despite these features, the multipole model and Hirshfeld atom refinement were chosen in this work because they are the most well established quantum crystallography methods currently available to deal with transition metals and different types of hydrogen contacts and offer a balance between accuracy and computational feasibility.

The quantum theory of atoms in molecules (QTAIM) (Bader, 1994 ▸), which can be applied to MM and HAR, provides a means to examine experimental data beyond distances and angles, deepening the information extracted from the experiment. It applies a partitioning scheme of molecules in open subsystems (atomic basins), generating, for instance, atomic charges through integrating electron densities in those atomic basins. Such analysis has been proven successful in studying chemical bonds in complexes containing transition metals, such as the Cr(CO)_6_, Fe(CO)_5_, and Ni(CO)_4_ (Farrugia & Evans, 2005 ▸) and short hydrogen bonds (Dos Santos *et al.*, 2012 ▸; Pinto *et al.*, 2023 ▸).

This study focuses on the application of multipolar and Hirshfeld atom refinements to the coordination compound tetra­aqua­bis­(hydrogenmaleato)iron(II) ([Fe(C_4_H_3_O_4_)_2_(H_2_O)_4_]) [hereafter abbreviated to FeHmal]. The complex presents distinct types of contacts, such as oxygen–metal coordination, intermolecular hydrogen bonds, and a short intramolecular hydrogen bond in the asymmetric unit. This makes the complex a good test for the ability of both refinements to provide a chemically reasonable model. Other structures containing the hydrogen maleate ligand have already been studied using quantum crystallography approaches, such as methyl­ammonium hydrogen maleate (Madsen *et al.*, 1998 ▸), l-phenyl­alanine hydrogen maleate (Woińska *et al.*, 2014 ▸), series of hydrogen maleate salts (Malaspina *et al.*, 2020 ▸; Malaspina *et al.*, 2021*b* ▸) and tetra­aqua­bis­(hydrogenmaleato)­nickel(II) (Pinto *et al.*, 2023 ▸). A neutron diffraction study for an analog compound tetra­aquabis(hydrogenmaleato)zinc(II) (Sequeira *et al.*, 1992 ▸) is available. This facilitates the analysis of bond lengths obtained from the herein-explored models with those determined using neutron diffraction.

Thus, two high-resolution X-ray diffraction measurements were carried out: one with higher completeness, *I*/σ(*I*), and *R*_int_ values, and another with the same resolution but with lower data quality. The aim was to evaluate the performance of the models in terms of their ability to obtain near-neutron diffraction bond lengths, appropriate anisotropic displacement parameters (ADPs), accurately modeled electron densities, and atomic charges under different experimental and modeling conditions. Also, the impact of the resolution limit was assessed by comparing the models with and without a cutoff in resolution. Table 1 ▸ presents a description of each model tested in this work.

## 2. Experimental

### 2.1. Synthesis and crystallization

Iron powder (0.28 g, 5 mmol) was heated to 353 K with an aqueous solution of maleic acid (1.74 g, 15 mmol) in 10 ml of water for 2 h until the iron was completely dissolved (Barman *et al.*, 2002 ▸). Crystals formed during the process were filtered, washed with a small amount of water, and dried in air. One suitable single crystal was selected for each X-ray diffraction experiment.

### 2.2. Data collection

High-resolution data collection (0.36 Å) was performed on an XtaLAB Synergy diffractometer, equipped with a HyPix detector, using Mo *K*α radiation (λ = 0.71073 Å), at 100 K. Two datasets were obtained. The first one [named Exp_slow] was collected more slowly (2.12 seconds per frame in low angles and 17.28 seconds per frame in high angles), resulting in higher *I*/σ(*I*) and lower *R*_int_ values. The other data set [named Exp_fast] was collected faster (0.72 seconds per frame in low angles and 5.75 seconds per frame in high angles). Data reductions of both experiments were carried out using *CrysAlisPro* (Rigaku Oxford Diffraction, 2019 ▸). The reflection data used for all refinements were scaled and merged in *SORTAV* (Blessing, 1987 ▸).

### 2.3. Independent atom model

The structure was solved by direct methods using *SHELXS* (Sheldrick, 1990 ▸) and refinement was performed in*SHELXL* (version 2018/3; Sheldrick, 2015 ▸) based on *F*^2^, both in *WinGX* (version 2021/3; Farrugia, 2012 ▸). All atoms, including hydrogen, were located in the Fourier difference map. Hydrogen atoms were refined with isotropic displacement parameters, while all the other atoms were refined anisotropically. The same procedure was carried out for Exp_slow and Exp_fast data sets.

### 2.4. Multipolar models

#### 2.4.1. Multipolar model for the Exp_slow data set (MM1)

Multipolar refinement based on the Hansen–Coppens model was performed in the *XDLSM* module of *XD2016* (Volkov *et al.*, 2016 ▸) for the Exp_slow data set. The scattering factors were taken from the Su–Coppens–Macchi databank (Su & Coppens, 1998 ▸; Macchi & Coppens, 2001 ▸). The merging process performed using *SORTAV* yielded 14020 reflections. Of these, 13896 with *I* > σ(*I*) were included in the refinement based on *F*^2^ (in order to better compare with the HAR1 procedure). The weighting scheme chosen was 1/σ^2^(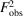) (for all MM-based refinements). The initial atomic coordinates and displacement parameters were taken from the final IAM refinement. Initially, this refinement was redone in *XD2016*, refining the positions and thermal motions of non-hydrogen atoms. Subsequently, kappa refinement was conducted. The refinement of the κ′ parameter for the iron atom was tested, but discarded as it did not result in an improvement of the model. Then, multipoles were refined, considering local symmetry of the atoms. All non-H atoms were refined up to the hexadecapole level. In contrast, hydrogen atoms were refined up to the quadrupolar level. Local symmetries were tested starting from the highest local symmetries to all atoms: *mmm* for Fe, *mm*2 to C atoms, and *m* to O atoms. Successive symmetry relaxations resulted in the best model with local symmetries: −1 to Fe, *m* to C atoms, and 1 to all O atoms except O6, for which the *m* symmetry was adequate. Symmetries *mm*2, *m*, and cylindrical were considered for H atoms. The final model considered cylindrical symmetry for all H atoms except H3*A*. For H3*A* (part of the short hydrogen bond), symmetry *m* was considered in the final model. *X*—H distances were initially constrained by the RESETBOND command based on neutron diffraction data for the analog complex but were freely refined in the subsequent refinement steps. Subsequently, ADPs for H atoms were estimated with *SHADE2.1* (Madsen, 2006 ▸) and inserted in the model, followed by a new refinement of the position of non-H atoms and multipoles of all the atoms, to fit the new ADPs generated. The isotropic extinction parameter was refined considering the model proposed by Becker & Coppens (1974 ▸). Topological properties were calculated in the *TOPXD* and *XDPROP* modules of *XD2016*. This model will be called MM1 hereafter.

#### 2.4.2. Multipolar model with a cutoff in reflections (MM1_cutoff)

An MM1-like refinement, with a resolution cutoff in *d* = 0.45 Å (resulting in 7330 merged reflections included in the refinement) was conducted, aiming to compare the effects of the resolution on the quality of refinement and to infer what features were added in the model exclusively by the higher-resolution part of the data. The subsequent procedures were carried out as outlined in Section 2.4. This model will be called MM1_cutoff.

#### 2.4.3. Multipolar model for the Exp_fast data set (MM2)

To access differences coming from experiments with the same resolution, but different quality data, a multipole refinement was employed for the Exp_fast data set. In this case, some adjustments were made in the refinement conditions, because the data quality was lower compared to that of Exp_slow. The refinement was carried out based on *F*, considering the reflections with *F* > 3σ(*F*). The remaining refinement procedure was essentially the same as in MM1. This model will be called MM2. Table S6 provides all chosen atom symmetries for MM-derived models. This model will be called MM2.

## 2.5. Hirshfeld atom refinement (HAR1)

HAR was executed using the *NoSpherA2* module in *Olex2*, using the same merged reflections as in MM1, starting from atomic coordinates and displacement parameters from the IAM refinement. The weighting scheme was 1/σ^2^(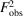) + 0.0065*P*)^2^ + 0.0083*P*], where *P* = (*F*_o_^2^ + 2*F*_c_^2^)/3 and the extinction parameter was refined. Quantum mechanical calculations were performed using *ORCA 5.0*, with the PBE0 functional and cc-pVTZ basis set, and assuming a vacuum environment. Multiplicities equal to 5 (high-spin model) and 1 (low-spin model) were tested for the Fe atom. The refinement was conducted based on *F*^2^. Positions and ADPs of all atoms were refined without any constraints or restraints.

The multiplicity 5 model proved to be more suitable, as it exhibited better statistical parameters [*R*(*F*) = 0.0176, *wR*(*F*^2^) = 0.0478 and *S* = 1.1446] compared with *R*(*F*) = 0.0223, *wR*(*F*^2^) = 0.0556 and *S* = 1.0625 for the multiplicity 1 model) and more appropriate deformation and residual maps in the metal region, as can be seen in Fig. S5. This aligns with a recent study on experimental spin state determination of a similar complex (which presents the Fe coordinated by six water molecules) using Hirshfeld atom refinement investigated by Brüx *et al.* (2025 ▸).

Topological analysis was carried out on the molecular wavefunction in *Multiwfn* (Lu & Chen, 2012 ▸). This model will be called HAR1.

## 2.6. Hirshfeld atom refinement with a cutoff in reflections (HAR1_cutoff)

A HAR1-like refinement, with a resolution cutoff at *d* = 0.45 Å (resulting in the same 7330 merged reflections included in the MM1_cutoff refinement) was conducted, so that the effects of the resolution in the refinement could be compared. The subsequent procedures were carried out as outlined in Section 2.4.1. This model will be called HAR1_cutoff.

## 3. Results and discussion

### 3.1. Model comparison

This section compares models MM1 and HAR, all obtained from Exp_slow data.

#### 3.1.1. Figures of merit

The two models demonstrate significant improvements over traditional IAM in terms of figures of merit, as seen in Table 2 ▸. This improvement is expected, as both MM and HAR account for aspherical features that IAM cannot capture. MM1 shows lower *R* values, as expected due to the higher number of refined parameters, while HAR1 shows a better agreement in terms of the *S* value (although this parameter depends on the weighting scheme adopted, which was different). The two refinements still exhibit a non-negligible amount of unmodeled electron density (especially HAR1), as indicated by Δρ_max_ and Δρ_min_ values. These values can be attributed primarily to the high-resolution data used in the experiment, since at a high angle (where the scattering intensity is low), only the core electrons contribute significantly.

#### 3.1.2. Crystal structure

##### 3.1.2.1. Molecular geometry

**Figure 1:**
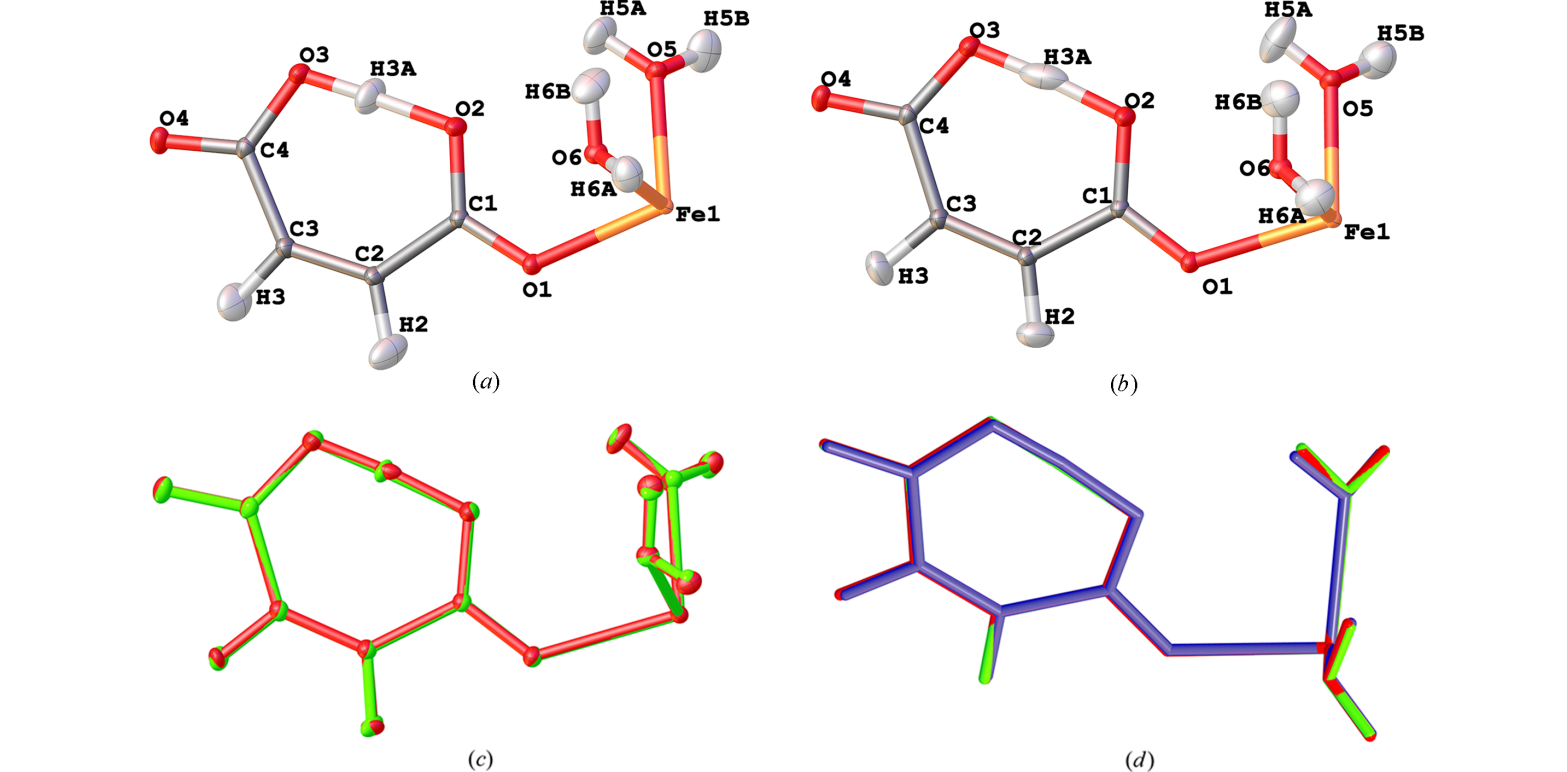
FeHmal crystal structures generated by (*a*) MM1, (*b*) HAR anisotropic atomic displacement ellipsoids are drawn at the 50% probability level. (*c*) Overlap of the structures of FeHmal by MM1 (green), and HAR1 (red), showing the ellipsoids. (*d*) Overlap of the structures of FeHmal by MM1 (green), HAR1 (red), and the zinc analog complex by neutron diffraction - MALAQZ03 (blue) (alignment RMSD = 0.023 Å).

A view of the two molecular structures with displacement ellipsoids can be seen in Fig. 1 ▸(*a*) for MM1 and Fig. 1(*b*) for HAR1, in which it is possible to see that the main visual differences between the models are in the ADPs of the H atoms. Fig. 1 ▸(*d*) presents an overlap of these models and, additionally, the structure obtained from neutron diffraction of the zinc analog complex (MALAQZ03; Sequeira *et al.*, 1992 ▸), in which the main difference lies in the O—H bond lengths of the water molecules. These variations may impact the interpretation of subsequently derived structural and electronic properties.

##### 3.1.2.2. Hydrogen bonding

Table 3 ▸ provides a comparative analysis of hydrogen bond distances and bond angles obtained from MM1, HAR1, and MALAQZ03 data. Accurate H-atom placement is crucial, as even slight variations can affect hydrogen-bond geometry and, consequently, the arrangement of atoms in the crystal. In general, *D*–H and H⋯*A* distances vary significantly among the models but remain close to the ones obtained from the neutron diffraction ones of the analog compound. The root-mean-square distance (RMSD) between the *D*–H distances refined in MM1 and the neutron distances is 0.0238 Å and the RMSD between the refined distances in HAR1 and the neutron distances is 0.0212 Å, showing that HAR1 performs slightly better in this sense.

Gilli & Gilli (2000 ▸) classified the hydrogen maleate ion as a negative charge-assisted hydrogen bond [(−)CAHB]. In their study, neutron diffraction data revealed an interdependence between the values of *d*(O—H) and *d*(H⋯O), indicating that strong O—H⋯O bonds tend to approximate these two distances to 1.20 Å, with *d*(O⋯O) ≅ 2.40 Å, in such a way that the H atom is in the electron densities of both O atoms. For the O3—H3*A*⋯O2 contact (the short intramolecular hydrogen bond), the *D*–H (and H⋯*A*) distance varies slightly between the methods, with MM1 providing lower *D*–H (and higher H⋯*A*) distances and HAR providing higher *D*–H (and lower H⋯*A*) distances. The two models result in O3⋯O2 distances lower than 2.42 Å, aligned with the classification as (−)CAHB and with the observation by Madsen *et al.* (1998 ▸) for the uncoordinated anion (although this anion contains a mirror plane not contained in the ligand coordinated to Fe). The asymmetry of the short hydrogen bonding is associated with the metal–ligand coordination as pointed out by Pinto *et al.* (2023 ▸).

More significant differences in distances and angles are observed for the hydrogen bonds involving water molecules. Both herein-presented models, particularly MM1, derive O5—H5*A* and H5*A*⋯O2 distances substantially longer than the distance obtained from neutron diffraction. In different ways, both models, but especially MM1, underestimate the O5—H5*B* bond length, although it does not deviate much from the reference concerning the other contacts involving this atom.

Regarding the distances from O6 to the H atoms to which it is bonded, HAR1 performs better, although MM1 gives the most consistent H6*A*⋯O4 and H6*B*⋯O1 distances. Finally, both models underestimate the *D*⋯*A* distances, except in the case of O2⋯O5, where it is overestimated, and in the case of O2⋯O3, where both models delivered values very close to those of neutron diffraction. Additional bond distances and angles are shown in Table S3.

**Figure 2:**
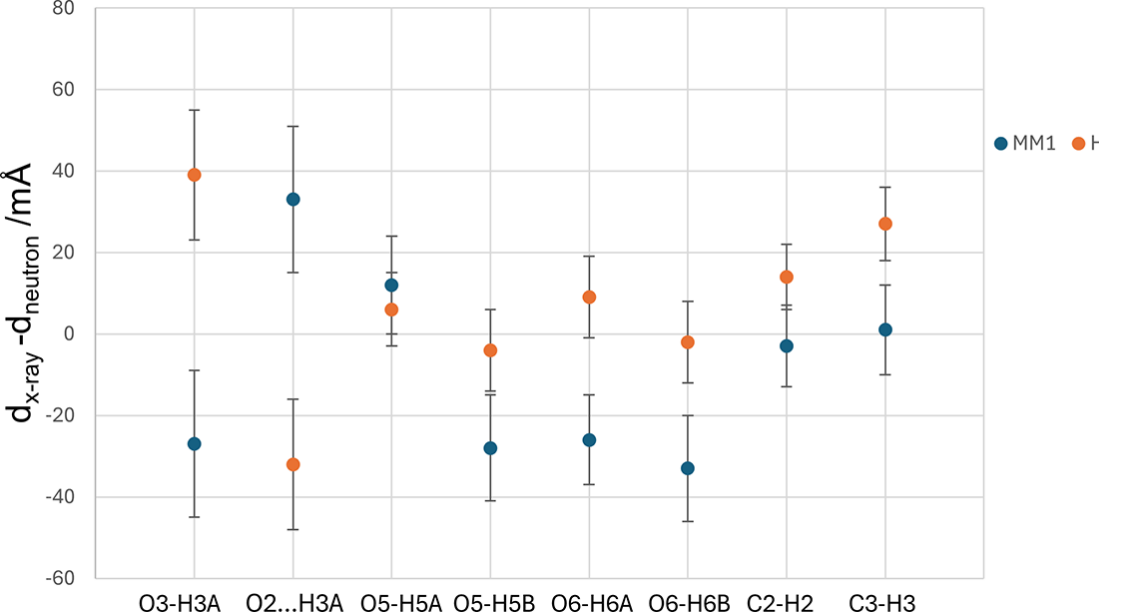
Differences between the X-ray and neutron diffraction bond distances for bonds containing hydrogen for the MM1 and HAR1 models. Error bars are the uncertainties of the distances derived by each model.

To better understand the variations in the distances obtained by the refinements, the graph in Fig. 2 ▸ shows these variations related to O–H and C–H distances. Fig. 2 ▸ illustrates the differences between bond lengths derived from X-ray and neutron diffraction for all bonds containing H atoms. In most O–H connections, MM1 tends to underestimate [something also observed by Pinto *et al.* (2023 ▸) for the nickel analog complex] the distances, except for O2⋯H3*A* and O5—H5*A*, where it overestimates. This model provides C–H distances very close to those from neutron diffraction. The HAR1 model displays an opposite trend, overestimating most bond lengths except for O2⋯H3*A*, which it underestimates, and O5—H5*B* and O6—H6*B*, where it provides values very close to those obtained from neutron diffraction.

Woińska *et al.* (2014 ▸) evaluated the capabilities of HAR in modeling the interactions in l-phenyl­alaninium hydrogen maleate, especially the short hydrogen bond. The results were compared to those obtained from IAM, MM, TAAM, and neutron diffraction data. The results showed that HAR produced the most accurate electron density model and the closest match to neutron diffraction results. Malaspina *et al.* (2020 ▸) investigated the hydrogen maleate anion stabilized by different cations using HAR and demonstrated that accurate and precise hydrogen-atom positions in short O—H⋯O hydrogen bonds can be achieved, whether isotropic displacement parameters are used, or ADPs are refined or estimated through *SHADE*. In the present study, HAR with free refinement of the ADPs was applied to the coordinated anion, showing potential for deriving *X*–H distances for compounds containing short hydrogen bonds coordinated to transition metals. Although the procedure occasionally overestimates or underestimates the bond distances observed in the compound under investigation, no consistent pattern for this behavior has been identified.

Olovsson *et al.* (1984 ▸) used three neutron-derived geometries of hydrogen maleate and one hydrogen chloro­maleate structure to derive a correlation between the O—H and O⋯H distances in the anion. Malaspina *et al.* (2017 ▸) extended their ideas using a larger data set of 17 neutron-diffraction structures involving the hydrogen maleate anion to derive a way to predict the hydrogen bond lengths starting from the position of the O atoms involved in the short contact. This method is expressed in equations (5) and (6). The O3—H3*A* and H3*A*⋯O2 distances obtained by applying these equations to the MM1 and HAR1 models are presented in Table 4 ▸ (with the approximation of the projection of the O⋯H interaction onto O⋯O as being the O⋯H distance and the projection of the O—H bond onto O⋯O as being the O–H distance, since the O—H⋯O bond angle is very close to 180°):
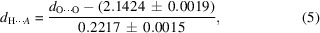

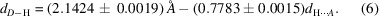
The distances are essentially the same in both models, which is expected given the similarity in the O3⋯O2 distance: 2.4186 (2) Å for MM1 and 2.4184 (2) Å for HAR1. When comparing the predicted values to those in Table 3, it is evident that the equation yields an O3—H3*A* bond length longer than in both models, while the H3*A*⋯O2 hydrogen bond distance is shorter.

##### 3.1.2.3. Anisotropic displacement parameters

In Figs. 1 ▸(*a*) and 1 ▸(*b*), the ellipsoids of non-H atoms are similar between the models, while those of H atoms differ, especially those of the H atoms of water molecules and the H atom involved in the short hydrogen bond (H3*A*). Fig. 1 ▸(*c*) shows an overlap of the two models (green-MM1, red-HAR1), with the structures rotated to minimize the RMSD between the two structures (alignment RMSD = 0.023 Å). In the HAR1, these ellipsoids appear larger and the H3*A* is noticeably stretched and flattened. This suggests that the procedure employed by generating displacement parameters in *NoSpherA2* captures a broader range of atomic motion for these H atoms. In contrast, ADPs from the MM1 model appear more uniform due to the use of *SHADE2.1*, since it estimates ADPs for H atoms considering a harmonic approximation rather than refining it. On the other hand, ADPs derived from HAR are directly refined against experimental data; therefore, they are subject to limitations related to data quality and experimental conditions.

The similarity index *S*_12_ (Whitten & Spackman, 2006 ▸), where *S*_12_ = 100(1 − *R*_12_) [*R*_12_ is derived in equation (7)] was employed to assess the differences in ADP values between the two models. *R*_12_ represents the overlap of the probability density functions (PDFs) corresponding to the analyzed ADP tensors. This index describes a percentage difference between the two PDFs (*p*_1_ and *p*_2_). *U*_1_ and *U*_2_ are the corresponding displacement tensors. Since the PDFs are normalized, when *U*_1_ = *U*_2_, *R*_12_ = 1.0 and consequently, there is no difference between the PDFs:
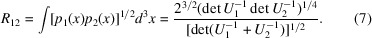


The combination of the figures of merit (FOM) based on the 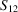 value, *R*_eigval_ (an index based on the magnitudes of corresponding eigenvalues) and the RMS error, is more informative since 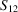 does not provide a direct measure of the relative orientation of the eigenvectors of the tensors (Sovago *et al.*, 2014 ▸). The three indices are combined according to equation (8),
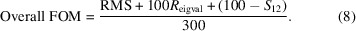
These indices were calculated using the SimADP routine in *WinGX*, where the two molecules being compared are rotated to minimize differences in their positional coordinates. *S*_12_, RMS, *R*_eigval_, and overall FOM for the overlapped atoms are presented in Table 5 ▸. Heavier atoms show significantly lower RMSD and *R*_eigval_ and *S*_12_ values closer to 100%, reflecting high similarity in the ADPs obtained by the two models. These statistics get worse for H atoms, showing that the main difference between the derivation of ADPs between the two models concerns the H atoms. The RMS_eigvec_ for H atoms is also notably higher, particularly for H3, H3*A*, H6*A* and H6*B*, with values exceeding 30°. The overall FOM for all atoms is consistently small, with lower values for heavier atoms and more elevated values for H atoms. The average *S*_12_ similarity index over all atoms in the molecule is 97.24%, indicating that the ADPs from the two models are highly consistent.

More insight into the ADPs can be obtained by comparing the results from the Hirshfeld rigid bond test (Hirshfeld, 1976 ▸), which evaluates the differences in amplitudes of mean square displacements (DMSDA) of the two atoms involved in a bond, serving to test whether the ADPs within a molecule are internally consistent. According to this test, for an adequate deconvolution of the vibration, this value should be as low as possible. As shown in Table 6 ▸, the results reveal a consistent trend where MM1 exhibits much lower DMSDA values for all *X*—H bonds. This trend indicates that the ADPs generated by *SHADE2.1* offer a more accurate representation of atomic vibrations. This outcome is expected since *SHADE2.1*’s methodology integrates neutron diffraction data and information about the bonded atom to derive ADPs for the H atoms.

#### 3.1.3. Residual and deformation electron density maps

**Figure 3:**
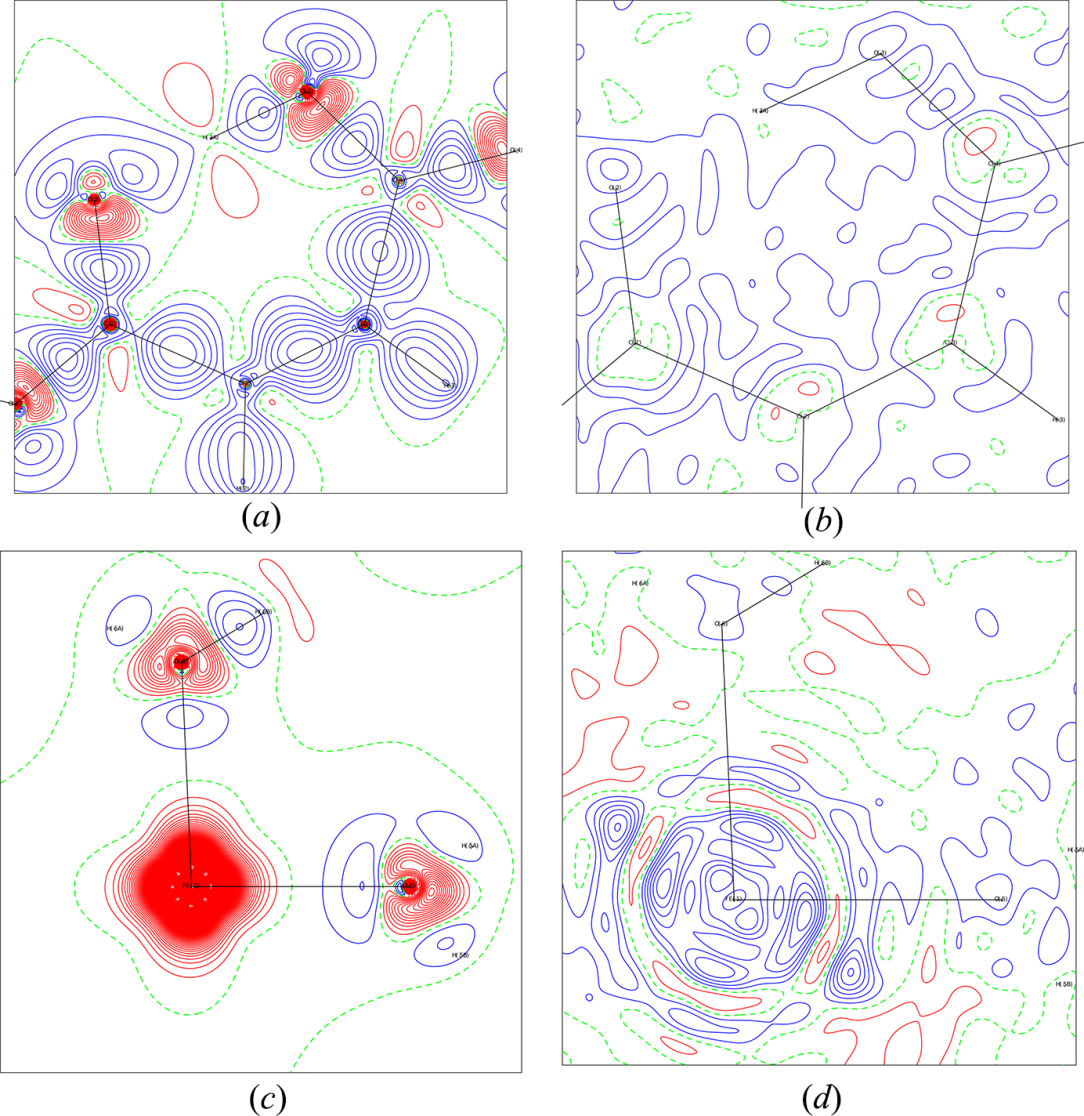
Deformation and residual maps for MM1: (*a*) deformation map in the hydrogen maleate plane, (*b*) residual map in the hydrogen maleate plane, (*c*) deformation map in Fe1–O5–O6 plane and (*d*) residual map in the Fe1–O5–O6 plane. Contours levels at 0.1 e Å^−^^3^, with positive contours in blue and negative contours in red.

**Figure 4:**
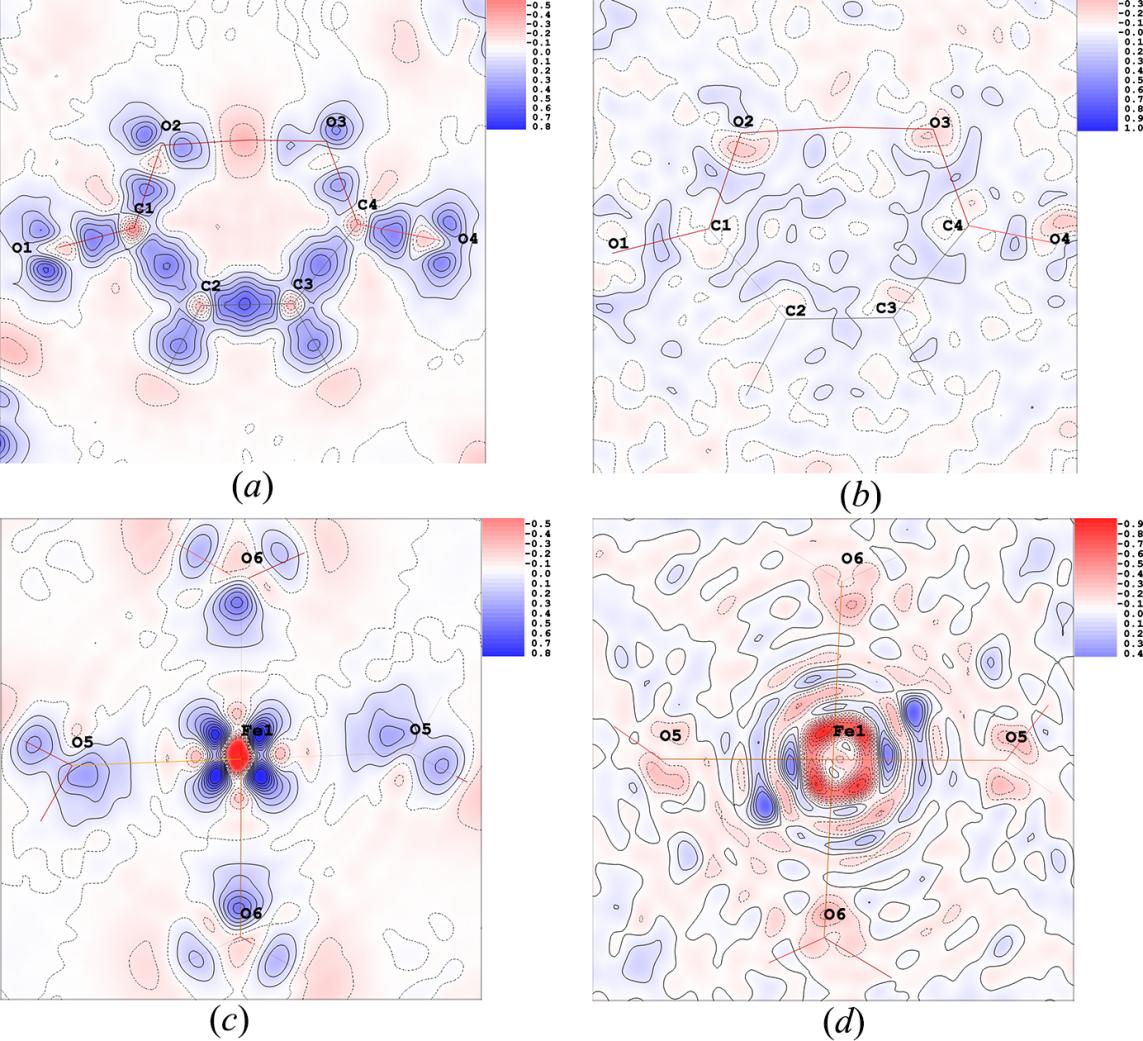
Deformation and residual maps for HAR1: (*a*) deformation map in the hydrogen maleate plane, (*b*) residual map in the hydrogen maleate plane, (*c*) deformation map in Fe1–O5–O6 plane and (*d*) residual map in the Fe1–O5–O6 plane. Contours levels at 0.1 e Å^−^^3^, with positive contours in blue and negative contours in red.

The final model quality can be assessed through residual and deformation maps. Fig. 3 ▸ (MM1), and Fig. 4 ▸ (HAR1) show these maps for the models explored in this work. All of them reveal electron density accumulated in chemical bonds and lone pairs in the water molecules and the hydrogen maleate ligand. None of the models show electron density overlap between iron and its bonded oxygen atoms, suggesting electrostatic character. Notably, in the region of Fe, the deformation map derived from multipole refinement displays a spherical appearance, while the HAR-derived map shows electron density accumulation between the ligands. All the models show a non-negligible number of residuals around Fe, due to the high resolution at which the X-ray diffraction experiment took place. Both models present low residues in the hydrogen maleate ligand region, especially MM1.

The residuals at O2 and O3 in the HAR model indicate signs of anharmonicity, which could potentially be modeled using Gram–Charlier coefficients. However, since this possibility was tested and discarded in MM1, and with the objective of maintaining the comparability between models, it was decided not to refine these coefficients in HAR1 either. Consequently, the electron density distributions around these O atoms are less accurately modeled, leading to noticeable residuals in their regions.

#### 3.1.4. Topological analysis

Both studied models allow the interpretation of properties derived from bond critical points (bcps). Table S2 presents the sum of distances from atoms to their bcps, electron density [ρ(**r**_bcp_)], Laplacian [∇^2^ρ(**r**_bcp_)], principal curvatures, and ellipticity at these points for diverse bonds and interactions in the compound. The values of λ_1_ and λ_2_ are negative, while λ_3_ is positive as expected for bond critical points

The analysis of the Laplacian reveals areas of charge concentration [negative ∇^2^ρ(**r**_bcp_)] and depletion [positive ∇^2^ρ(**r**_bcp_)]. The Laplacian values from the two models cannot be directly compared, as this property represents the second derivative of the electron density. Consequently, small variations in the electron density can result in significant changes in the Laplacian. However, analyzing the signs and orders of magnitude offers valuable insight into the chemical nature of the interactions. The interpretations of these properties are very similar in the two models.

Positive and small values of the Laplacian (the sum of λ_1_, λ_2_, and λ_3_) denote that atoms are connected electrostatically by closed-shell interactions, such as ionic bonds, as seen in Fe—O bonds across both models, consistent with crystal field theory. This also happens in all hydrogen bonds involving water molecules and carb­oxy­lic oxygens. Negative values of the Laplacian indicate open-shell interactions, with concentration of electron density in the internuclear regions, as in covalent bonds, which are evident in O—H bonds in water molecules and C—H bonds within the hydrogen maleate ligand in both models

Both models yield negative Laplacians for all C–C, C–H, O–H, and C–O interactions, indicating covalency. The water hydrogen atoms are involved in typical electrostatic hydrogen bonds (O—H⋯O), with one covalent (O—H) and one ionic (H⋯O) interaction, according to the observed Laplacian values. In the case of the short hydrogen bond (O3—H3*A*⋯O2), particularly, both models give negative Laplacian for both interactions, indicating that the hydrogen interacts covalently with both O atoms, inline with Pinto *et al.* (2023 ▸) for the analog complex and Madsen *et al.* (1998 ▸) for the uncoordinated ion. Particularly, the magnitude of these interactions differentiates this hydrogen bond from those involving water molecules, in which O—H bonds show largely negative Laplacians and H⋯O interactions have small and positive values, which indicates electrostatic interaction. There is a difference in the distance between the O and H atoms of the short hydrogen bond comparing the uncoordinated anion with the present work, since in the first, there is a plane of symmetry that causes the critical points to be found at the same distance (*ca* 0.30 Å) from the H/D atom, while in this work the distance from H3*A* to the bcp that connects O3—H3*A* is 0.234 Å (MM1) and 0.250 Å (HAR1) and the distance from H3*A* to the bcp that connects O2⋯H3*A* is 0.392 Å (MM1) and 0.327 Å (HAR1).

The values of ρ(**r**_bcp_) found here for C—C bonds are close to those found for the uncoordinated ion, despite the absence of the plane of symmetry in the ligand in the present study. The metal–ligand coordination seems to have some effect on the value of ρ(**r**_bcp_) for the C1—O1 bond, since for the uncoordinated anion this value is 2.92 e Å^−3^, for coordination with nickel it is 2.73 e Å^−3^ and in this work, the value is 2.618 e Å^−3^ (MM1) and 2.562 e Å^−3^ (HAR1).

The ellipticity value (ɛ) evaluates how cylindrical the bonds are, enabling to differentiate between single and double bonds. For both models, the C2—C3 bond has the greatest ɛ value of all covalent bonds, aligned to the expected double bond.

Espinosa *et al.* (1999 ▸) identified an exponential relationship between λ_3_ and the *d*(H⋯O) distance, which is reproduced here as equation (9),[Disp-formula fd9]
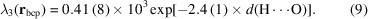
Considering the uncertainty of the values found applying this equation, the λ_3_ values for hydrogen bonds coming from MM1 and HAR1 in Table 4 ▸ align with the findings of Espinosa *et al.* (1999 ▸) and a comparison in given in Table 7 ▸. The differences arise from the fact that, in the present work, the hydrogen atoms had their positions freely refined whereas the equation was based on combined X-ray and neutron diffraction data.

**Figure 5:**
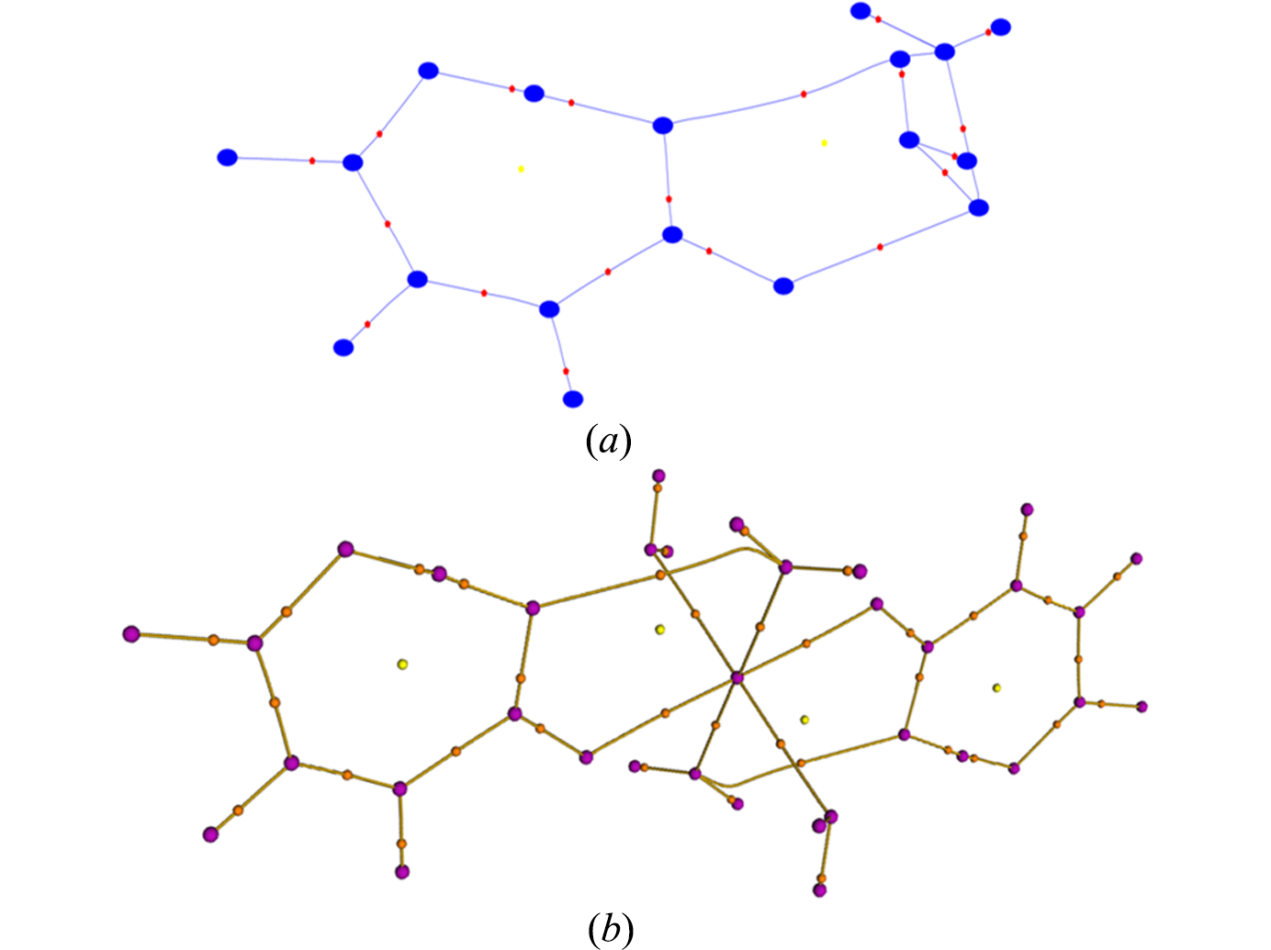
FeHmal molecular graphs (xdprop module) for (*a*) the asymmetric unit of MM1 and (*b*) the molecular unit of HAR1. (*a*) Blue dots are (3, +3) critical points, red dots (3, −1) critical points and yellow dots (3, +1) critical points, HAR1. (*b*) Purple dots are (3, +3) critical points, orange dots are (3, −1) critical points and yellow dots are (3, +1) critical points. The molecular graph generated for the HAR1 model presents the entire molecule since the calculations done in the HAR procedure consider the entire unit cell.

Fig. 5 ▸ presents the molecular graphs generated from the two models containing all critical points and bond paths. All models present virtually the same (3, −3) critical points due to nuclear positions, (3, −1) bond critical points, and (3, +1) ring critical points, one formed as a consequence of the short intramolecular hydrogen bond and the other due to the bond critical point generated by the O5—H5*A*⋯O2 interaction. The main difference is in the bond path formed by this interaction, for which HAR1 presents a steeper curve, while MM1 is straighter.

Abramov (1997 ▸) proposed a methodology to estimate the kinetic energy density, *G*(**r**), for closed-shell interactions in terms of ρ(**r**) and ∇ ^2^ρ(**r**) at the critical bonding point, given in equation (10),
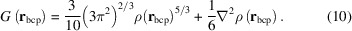
The Laplacian of the electron density is related to energy densities through equation (11), where *V*(**r**) is the potential energy density at point **r**:
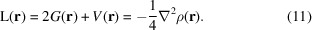
The electronic energy density *H*(**r**) can be calculated with equation (12),[Disp-formula fd12]
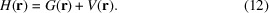
Espinosa and co-workers (2002 ▸) established that when |*V*(**r**_bcp_)|/*G*(**r**_bcp_) > 2, the interaction is classified as open-shell and when it is < 1, it is closed-shell. It is possible to see these properties for Fe—O bonds and hydrogen bonds within the explored models in Table S3.

In all models, the |*V*(**r**_bcp_)|/*G*(**r**_bcp_) ratio for Fe—O bonds is close to 1, which, together with *H*(**r**_bcp_) very close to zero, indicates a predominance of purely closed-shell interactions. The HAR1 model yields a value slightly greater than unity for the *V*(**r**_bcp_)|/*G*(**r**_bcp_) ratio for the Fe—O1 bond, suggesting a small covalence for all Fe—O bonds. Values of this ratio between 1 and 2 for the O4⋯H5*B* and O1⋯H6*B* intermolecular interactions in MM1 indicate that these contacts are in the transition region between closed-shell and shared-shell. O2⋯H5*A*, O3⋯H5*A*, and O4⋯H6*A* have values lower than unity for all models, suggesting nature totally electrostatic.

In both models, the O2⋯H3*A* interaction has a |*V*(**r**_bcp_)|/*G*(**r**_bcp_) ratio greater than 2 (significantly higher than the value found by Pinto *et al.* (2023 ▸) for the analog nickel complex using multipolar refinement), which, along with the negative Laplacian, the high value of ρ(**r**_bcp_), *G*(_bcp_)/ρ(**r**_bcp_) smaller than 1 and *H*(**r**_bcp_)/ρ(**r**_bcp_) smaller than 0, supports its classification as purely covalent bond by these models.

Table 8 ▸ presents atomic charges calculated using QTAIM for both models. All of them yield very close to zero total charges, consistent with the real crystal expected charge. In the case of MM1, the total value of the sum of the charges is 0.01 e.

Both models lead to charges with chemical sense since they lead to O atoms (more electronegative) with more negative charges and C atoms (more electropositive, and, in addition, bonded to oxygens) with a positive charge.

MM1 and HAR1 models differ significantly in the charge of the iron atom, with the former being closer to the chemically expected +2. The MM1 model also gives a bigger charge for H3*A*. There is also a considerable difference between the values from the two models for the sum of the charges of the hydrogen maleate ligand, although both derive values close to −1. Additionally, the MM1 model gives a bigger charge and smaller volume for H3*A*, consistent with its strong interaction with both oxygens in the short hydrogen bond. The MM-derived methods use the Lagrangian parameter to evaluate the integration, with 〈|*L*(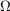)|〉 = 6.4 × 10^−4^ a.u.

HAR1 volumes differ substantially from those of MM1 and from the total unit cell volume (experimental value of 318.456 (5) Å^3^. This difference is due to the molecular wavefunction being calculated in a vacuum environment, which does not account for the crystal packing.

### 3.2. On the importance of good data collection

#### 3.2.1. MM1 *versus* MM1_cutoff

The importance of high-resolution measurements in charge density studies is well known. The data collection up to high angles is necessary because the core electrons scatter at larger values, for which the atomic scattering factors decrease significantly (Woolfson, 1997 ▸), and to adequately model the charge density, accounting for subtle effects such as deviations from sphericity in the electron distribution. The main experiment in this work (Exp_slow) was carried out up to a resolution of *d* = 0.36 Å, and, thus, accounts for a deep electron distribution in the atoms of the complex, especially in the iron region. A refinement similar to the MM1 but using only the reflections that fall at resolution cutoff at *d* = 0.45 Å was conducted to assess the impact of resolution on refinement quality.

**Figure 6:**
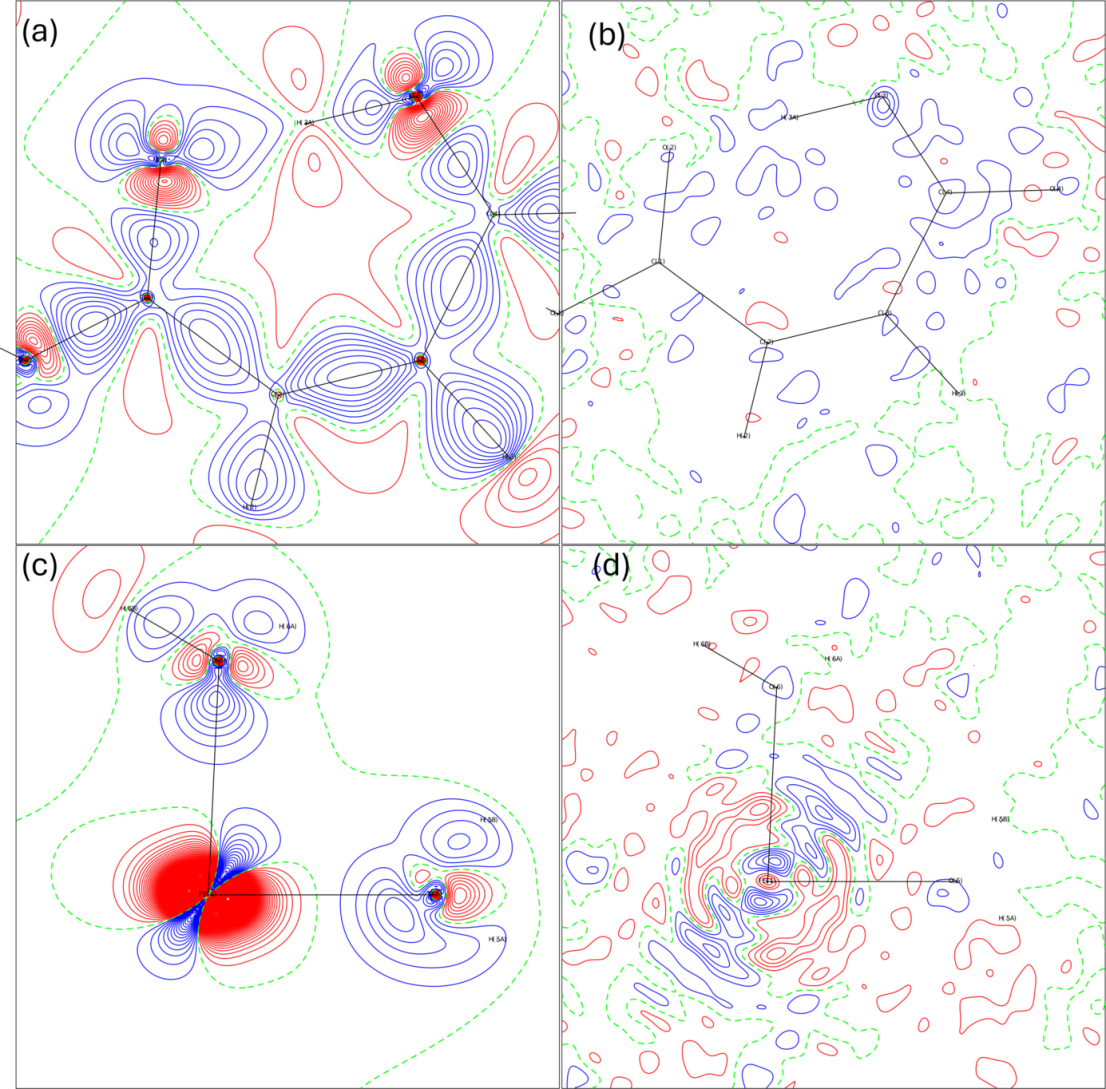
Deformation and residual maps for MM1_cutoff: (*a*) deformation map in the hydrogen maleate plane, (*b*) residual map in the hydrogen maleate plane, (*c*) deformation map in Fe1–O5–O6 plane and (*d*) residual map in the Fe1–O5–O6. Contours levels at 0.1 e Å^−^^3^, with positive contours in blue and negative contours in red.

Fig. 6 ▸ exhibits deformation and residual maps for MM1_cutoff. It is visible that the electron density in this model is more spread in the bond regions and is much less defined than in the higher-resolution model. There are, naturally fewer residuals compared to MM1, due to the exclusion of high angle scattering data, with the main ones remaining around the metal region.

Iron atomic charge and volume were calculated through QTAIM for this model, yielding a value of 1.57 e and volume of 9.24 Å^3^ (Lagrangian of 9.76 × 10^−5^). This charge is lower than the value obtained from the full-resolution MM1 model (2.23 e). This difference highlights the impact of the resolution on the atomic charges, especially for the Fe cation. Among all atoms of the structure, Fe is the main contributor to scattering in high resolution. The number of core electrons in Fe is much higher than the number of any other atom in the structure.

#### 3.2.2. HAR1 versus HAR_cutoff

**Figure 7:**
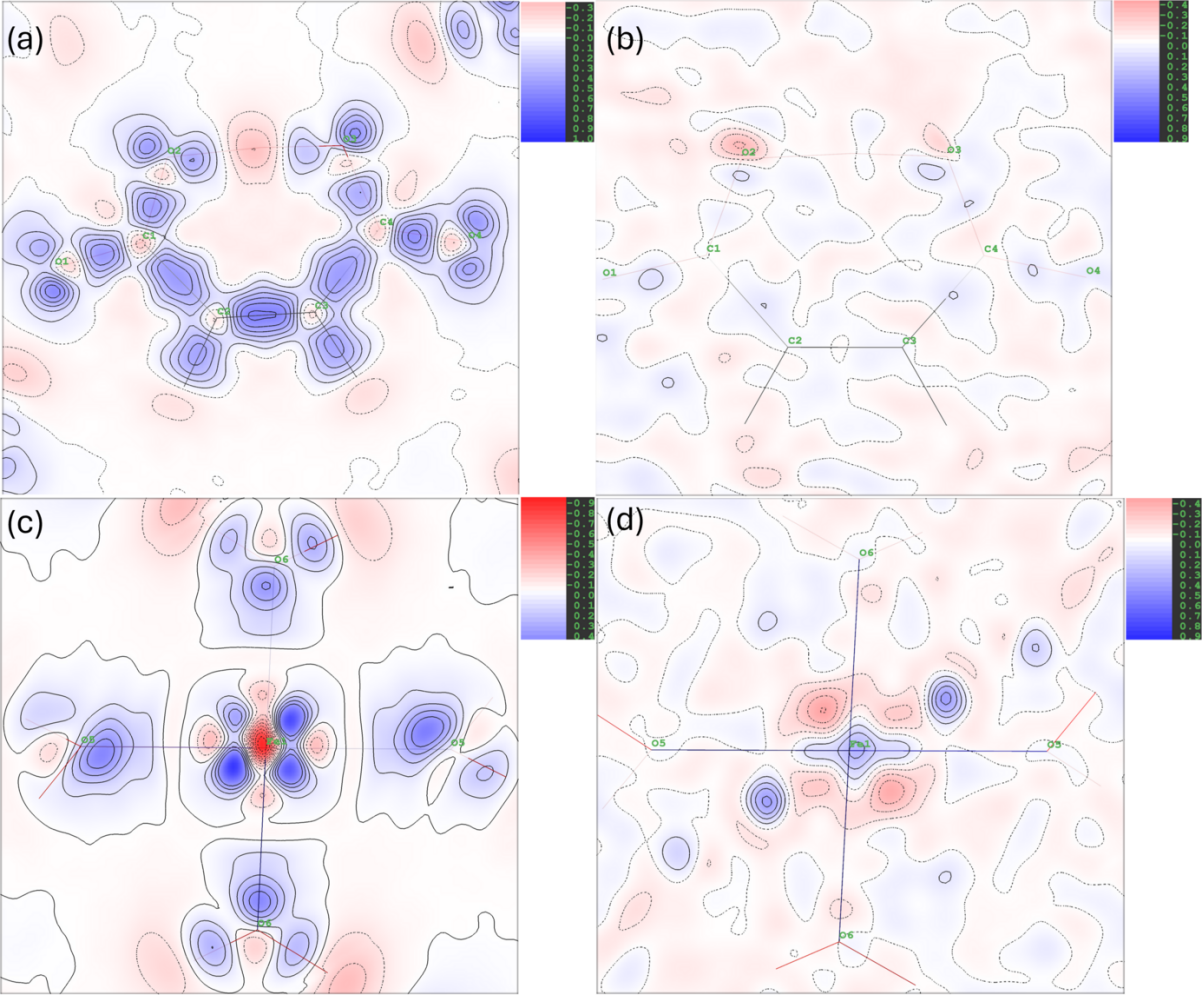
Deformation and residual maps for HAR1_cutoff: (*a*) deformation map in the hydrogen maleate plane, (*b*) residual map in the hydrogen maleate plane, (*c*) deformation map in Fe1–O5–O6 plane and (*d*) residual map in the Fe1–O5–O6 plane. Contour level at 0.1 e Å^−^^3^, with positive contours in blue and negative contours in red.

The HAR1_cutoff refinements were conducted under nearly identical conditions to HAR1. Analogously to MM1_cutoff, there are, naturally fewer residuals (Fig. 7 ▸) compared to HAR1, due to the exclusion of high-angle scattering data. The properties such as distances and bond angles of this refinement are extremely similar to those obtained in HAR1 and will not be detailed here. Similar results performing Hirshfeld atom refinement at different resolutions were also found by Pinto *et al.* (2023 ▸) for the nickel analog complex.

#### 3.2.3. MM1 *versus* MM2

The statistical parameters in Table S4 provide a view of the data quality across resolution ranges for the Exp_slow and Exp_fast., the data used for MM1 and MM2, respectively. The average redundancy decreases as resolution increases since higher-resolution data are more challenging to collect. The data collection maintains nearly 100% completeness throughout both experiments, with a slight decrease in the two final ranges. In both cases, there is a drop in the mean *F*^2^/σ(*F*^2^) across the resolution ranges since reflection intensities become weaker and more difficult to distinguish from the noise at higher resolutions. However, for the Exp_slow data collection, this drop is less pronounced than for Exp_fast. Consistently, the *R*_int_ values naturally increase within the ranges, reflecting the challenge of accurately measuring and merging high-resolution data, with better performance for Exp_slow, presenting the smallest increase, reflecting in the better subsequent statistical parameter values and interpretation of electron density distributions.

Table 9 ▸ presents a comparative view of the iron orbital population for three multipolar models explored in this work. Model MM1 shows somehow similar occupations for the three 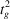 orbitals (*d*_*xz*_, *d*_*xy*_, and *d*_*yz*_) and for the two *e*_*g*_ orbitals (*d*_*z*^2^_ and *d*_*x*^2^–*y*^2^_) in accordance with the spherical appearance of the electron distribution around the iron seen in Fig. 3 ▸.

**Figure 8:**
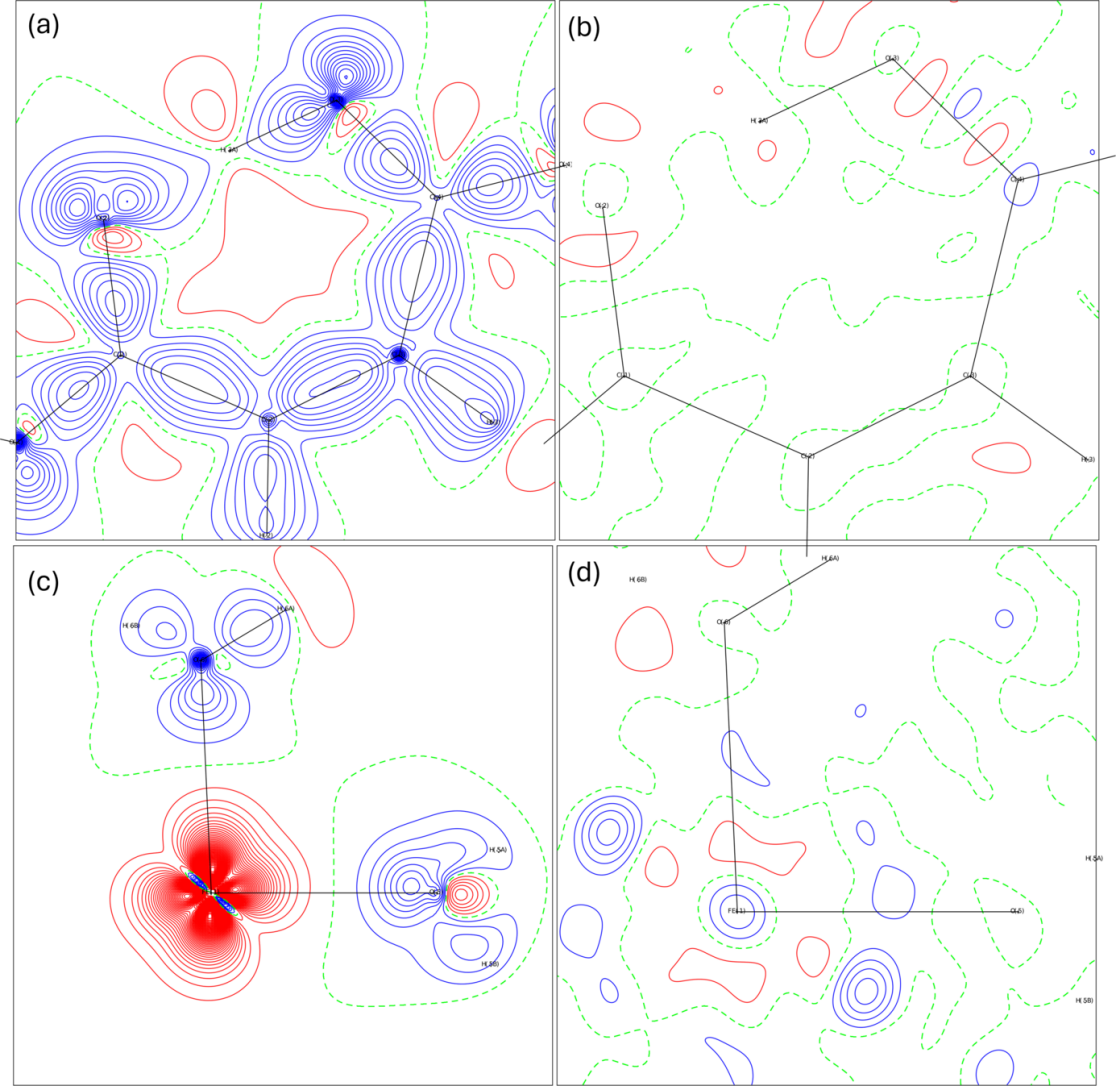
Deformation and residual maps for MM2 with contour level at 0.1 e Å^−3^: (*a*) deformation map in the hydrogen maleate plane, (*b*) residual map in the maleate plane, (*c*) deformation map in Fe1–O5–O6 plane and (*d*) residual map in the Fe1–O5–O6 Contours levels at 0.1 e Å^−^^3^, with positive contours in blue and negative contours in red.

Regarding MM2 refinement, orbital populations show significant variations relative to MM1, as can also be inferred by their respective deformation maps in the Fe1–O5–O6 plane [Fig. 8 ▸(*c*)], which shows electron accumulation between the ligands in the case of MM2 and does not show in MM1. Notably, for the MM2 model, the *d*_*z*^2^_ orbital has the second highest occupancy, but for the Exp_slow-derived models, this orbital has the lowest occupancy of all.

Interestingly, the total *d* population obtained from model MM1 is equal to 5.94, basically leading to the expected Fe^II^ ion occupation of d orbitals, in good agreement with the charge obtained using the QTAIM integration (Table 8 ▸).

Table S5 presents atomic charges and volumes calculated by QTAIM for the MM2 model. The mean Lagrangian is 〈|*L*(Ω)|〉 = 5.76 × 10^−4^ a.u. and the volumes are very close to those obtained by the other multipolar-derived refinements. However, this model differs significantly from the others regarding the charge of the Fe atom, presenting the smaller one shown in this work, which is similar to what Pinto and co-workers found (0.809 e) for the Ni atom in an analog complex.

The total charge of the hydrogen maleate ligand also shows significant variation compared to the other models presented, with a value approximately half of that observed in MM1, for instance.

These data show that faster acquisition with poorer statistics can yield results that differ significantly from those obtained with better measurements, potentially leading to less accurate chemical results.

## 4. Conclusion

Tetra­aqua­bis­(hydrogenmaleato)iron(II) was studied in a high-resolution single-crystal X-ray diffraction experiment and Hansen–Coppens MM and HAR were employed. Both models represented an improvement in the figures of merit and chemical description of the compound in comparison to IAM.

Topological analysis based on the QTAIM was performed for both models, which allowed a quantitative description of the chemical bonds of the compound. The covalent and electrostatic characters of the bonds were elucidated. The analysis allowed the conclusion that the compound presents interactions with distinct characters. The short intramolecular hydrogen bond has a special character since hydrogen interacts covalently with the two O atoms. Atomic charges were calculated for the models, and all were chemically consistent.

The multipole model yields highly satisfactory results for all the properties analyzed. However, its performance is significantly influenced by variations in data collection strategies and the resolution cutoff. In contrast, HAR results are less influenced by data quality, yielding similar bond distances and other properties under varying conditions. However, the ADPs derived for hydrogen atoms using this model, particularly for the one involved in the short hydrogen bond, exhibit notable issues. The multipole model, when used in conjunction with *SHADE2.1*, resulted in very accurate anisotropic displacement parameters, as expected, due to the methodology used.

Overall, these models and the topological analysis provided deeper insights into the electron density distribution of the compound and the nature of the hydrogen bonds within the compound.

## Supplementary Material

Crystal structure: contains datablock(s) I, MM1cutoff, HAR, HARcutoff, MM2. DOI: 10.1107/S2052520625003403/woz5002sup1.cif

Structure factors: contains datablock(s) I. DOI: 10.1107/S2052520625003403/woz5002sup2.hkl

Structure factors: contains datablock(s) . DOI: 10.1107/S2052520625003403/woz5002sup3.hkl

Structure factors: contains datablock(s) . DOI: 10.1107/S2052520625003403/woz5002sup4.hkl

Tables S1-S7, Figs. S1-S5. DOI: 10.1107/S2052520625003403/woz5002sup5.pdf

CCDC references: 2444203, 2444758, 2444759, 2444760, 2444761

## Figures and Tables

**Table 1:** Description of each model

Model	Description
MM1	Multipolar refinement for a high-resolution (*d* = 0.36 Å) measurement, employing *SHADE2.1* for generation of hydrogen ADPs.
HAR1	Hirshfeld atom refinement, in *NoSpherA2* module in *Olex2*, with the PBE0 functional and cc-pVTZ basis set, refining atomic positions and ADPs without constraints.
HAR1_cutoff	Same methodology as HAR1 but with a reflection cutoff at *d* = 0.45 Å.
MM1_cutoff	Same methodology as MM1 but with a reflection cutoff at *d* = 0.45 Å.
MM2	Multipolar refinement for high-resolution (*d* = 0.36 Å) data, collected under faster conditions than in MM1, resulting in lower completeness, redundancy, and higher *R*_int_ values.

**Table 2:** Refinement statistics for the multipolar model and Hirshfeld atom refinement of tetra­aqua­bis­(hydrogeno­maleato)iron(II)

	IAM	MM1	HAR1
*R*(*F*)	0.0209	0.0162	0.0176
*wR*(*F*^2^)	0.0570	0.0454	0.0478
*S*	1.058	1.4093	1.1446
No. of independent reflections [*I* > σ(*I*)]	14020, 13896	13896	13896
No. of parameters	126	346	161
Δρ_max_, Δρ_min_ (e Å^−3^)	0.90, −1.10	0.60, −0.29	0.50, −0.73

**Table 3:** Hydrogen-bond geometry (Å, °) obtained from MM1 (1st line), HAR1 (2nd line), and neutron diffraction from the zinc analog complex (3rd line)

*D*—H⋯*A*	*D*—H	H⋯*A*	*D*⋯*A*	*D*—H⋯*A*
O3—H3*A*⋯O2	1.070 (18)	1.349 (18)	2.4186 (2)	177.7 (12)
	1.136 (16)	1.284 (16)	2.4184 (2)	175.6 (11)
	1.097 (7)	1.316 (5)	2.410 (4)	174.7 (4)
O5—H5*A*⋯O2	0.941 (12)	2.391 (13)	2.9014 (2)	113.8 (9)
	0.935 (9)	2.380 (13)	2.9014 (2)	114.9 (10)
	0.929 (8)	2.253 (5)	2.792 (3)	122.2 (4)
O5—H5*A*⋯O3^i^	0.941 (12)	1.996 (12)	2.8554 (2)	151.0 (11)
	0.935 (9)	2.008 (11)	2.8560 (2)	150.0 (12)
	0.929 (8)	2.108 (6)	2.888 (3)	140.8 (4)
O5—H5*B*⋯O4^ii^	0.929 (13)	1.840 (12)	2.7555 (2)	168.3 (11)
	0.953 (10)	1.814 (11)	2.7555 (2)	169.3 (11)
	0.957 (9)	1.848 (7)	2.789 (4)	167.4 (4)
O6—H6*A*⋯O4^iii^	0.924 (11)	1.927 (11)	2.8145 (2)	160.3 (11)
	0.959 (10)	1.885 (10)	2.8150 (2)	162.9 (10)
	0.950 (8)	1.949 (6)	2.869 (3)	162.4 (4)
O6—H6*B*⋯O1^iv^	0.932 (13)	1.885 (13)	2.8154 (2)	175.3 (10)
	0.963 (10)	1.853 (10)	2.8153 (2)	177.9 (10)
	0.965 (7)	1.899 (5)	2.861 (3)	174.9 (3)

Symmetry codes: (i) 1 − *x*, 1 − *y*, 1 − *z*; (ii) *x*, *y* − 1, *z* − 1; (iii) *x*, *y*, *z* − 1; (iv) *x*, *y*, *z* − 1.

**Table 4:** O3—H3*A* and H3*A*⋯O2 hydrogen bond distances (Å) found for FeHmal, determined using equations (5) and (6) MM1 (1st line), HAR1 (2nd line).

O3—H3*A*	H3*A*⋯O2
1.173 (11)	1.246 (12)
1.173 (11)	1.245 (12)

**Table 5:** RMSD (°) of principal axes of the orthogonalized *U*_*ij*_ tensors (degrees), *R*_eigval_, *S*_12_ index (%), and overall figure of merit (FOM) for atomic positions obtained for all atoms upon overlap of the structures obtained by MM1 and HAR1

	RMSD	*R*_eigval_	*S*_12_	Overall FOM
Fe1	1.73	0.003	99.96	0.007
O1	0.42	0.009	99.97	0.005
O2	6.45	0.012	99.98	0.026
O3	0.89	0.009	99.98	0.006
O4	0.67	0.009	99.98	0.005
O5	1.26	0.006	99.98	0.006
O6	4.41	0.004	99.97	0.016
C1	3.85	0.011	99.93	0.017
C2	1.11	0.011	99.94	0.008
C3	0.11	0.013	99.94	0.005
C4	0.58	0.007	99.94	0.004
H2	26.84	0.249	92.94	0.196
H3	34.75	0.074	92.98	0.164
H3*A*	35.23	0.078	83.62	0.198
H5*A*	29.94	0.114	95.18	0.154
H5*B*	25.92	0.199	96.50	0.164
H6*A*	40.27	0.064	98.19	0.162
H6*B*	35.16	0.337	91.35	0.258

**Table 6:** DMSDA values (1 × 10^−4^ Å^2^) in the O—H and C—H bonds (Å) from the evaluated models: MM1 (1st line), HAR1 (2nd line)

Atom 1	Atom 2	Distance	DMSDA	Atom 1	Atom 2	Distance	DMSDA
O6	H6*A*	0.924	69	O3	H3*A*	1.070	49
		0.959	107			1.136	418
O6	H6*B*	0.932	65	O2	H3*A*	1.349	46
		0.963	207			1.284	406
O5	H5*A*	0.941	51	C2	H2	1.067	56
		0.935	68			1.084	65
O5	H5*B*	0.929	60	C3	H3	1.081	59
		0.952	177			1.107	63

**Table 7:** Values of λ_3_ found in this work and using equation (9) MM1 (1st line), HAR1 (2nd line).

H⋯O	This work	Espinosa *et al.*	H⋯O	This work	Espinosa *et al.*
O2⋯H3*A*	15.297	16.1 (3.9)	O4⋯H5*B*^ii^	4.859	5.0 (1.3)
	17.737	18.8 (4.5)			5.3 (1.4)
O2⋯H5*A*	1.700	1.3 (4)	O4⋯H6*A*^iii^	4.206	4.0 (1.1)
	1.518	1.4 (4)			4.4 (1.2)
O3⋯H5*A*^i^	3.330	3.4 (1.0)	O1⋯H6*B*^iii^	4.424	4.4 (1.2)
		3.3 (9)			4.8 (1.3)

Symmetry codes: (i) 1 − *x*, 1 − *y*, 1 − *z*; (ii) *x*, *y* − 1, *z* − 1; (iii) *x*, *y*, *z* − 1.

**Table 8:** Atomic charges (e) and volumes (Å^3^) from QTAIM for MM1 (1st line), HAR1 (2nd line) There are two values in the HAR1† line because the HAR procedure calculates the wavefunction for both asymmetric units of the unit cell. Total_Hmal_ refers to the atoms in the hydrogen maleate anion; Total_w5_ denotes the water molecule composed of O5, H5*A*, and H5*B*; and Total_w6_ corresponds to the water molecule formed by O6, H6*A*, and H6*B*.

Atom	*q*_Ω_	*V*_Ω_	Atom	*q*_Ω_	*V*_Ω_
Fe1	2.03	10.05	H3*A*	0.67	0.94
	1.53	10.00		0.65	1.23
O1	−1.38	15.42	Total_Hmal_	−1.50	113.91
	−1.27	17.40		−0.87	122.73
O2	−1.35	16.41	O5	−1.07	15.91
	−1.25	17.35		−1.25	17.99
O3	−1.28	15.86	H5*A*	0.69	1.56
	−1.23	17.74		0.67	2.48
O4	−1.34	16.90	H5*B*	0.65	1.56
	−1.16	20.05		0.63	2.85
C1	1.87	4.90	Total_w5_	0.27	19.03
	1.67	5.29		0.05	23.32
C2	−0.22	11.49	O6	−1.00	16.89
	−0.07	12.58		−1.21	18.56
C3	−0.43	12.38	H6*A*	0.60	1.89
	−0.04	12.18		0.63	2.92
C4	1.85	5.40	H6*B*	0.62	1.75
	1.70	5.31		0.64	2.86
H2	0.03	7.47	Total_w6_	0.22	20.53
	0.05	6.85		0.06	24.34
H3	0.08	6.73	Unit cell	0.01	316.99
	0.09	6.76		0.00	350.79

† Although *Multiwfn* calculates the topological properties ignoring symmetry (for each pair of atoms, two values are calculated), only one value is shown for each pair of atoms in the HAR1 line because the values are numerically identical after rounding.

**Table 9:** Iron orbital populations (e, %) from the MM1, MM1_cutoff, and MM2 models

	MM1		MM1_cutoff		MM2	
Orbital	e	%	e	%	e	%
*d*_*xz*_	1.39	23.5	1.54	24.3	1.69	24.4
*d*_*xy*_	1.31	22.1	1.44	22.7	1.32	19.4
*d*_*yz*_	1.19	20.1	1.26	19.9	1.38	20.2
*d*_*x*^2^–*y*^2^_	1.12	18.8	1.13	17.9	0.91	13.5
*d*_*z* ^2^_	0.93	15.6	0.96	15.2	1.51	22.2
Total	5.94		6.33		6.83	
