# Supplementary material for: Multipolar model and Hirshfeld atom refinement of tetra­aqua­bis­(hydrogenmaleato)iron(II)
Source: Acta Crystallogr B Struct Sci Cryst Eng Mater. 2025 May 9;81(Pt 3):350–62. doi: 10.1107/S2052520625003403 (PMC12147936; doi:10.1107/S2052520625003403)
Supplement: Supplementary file 5 [file b-81-00350-sup5.pdf]

STRUCTURAL SCIENCE
CRYSTAL ENGINEERING
MATERIALS

**Volume 81 (2025)**

**Supporting information for article:**

# Multipolar Model and Hirshfeld Atom Refinement of tetraaquabis-(hydrogenmaleato)iron(II)

**Hellen Ferreira Guimarães and Bernardo Lages Rodrigues**

**Table S1**

Crystallographic data of FeHmal obtained from Exp_slow and Exp_fast.

| | Exp_slow | Exp_fast |
|---|---|---|
| *Crystal data* | | |
| Chemical formula | $C_8FeH_{14}O_{12}$ | $C_8FeH_{14}O_{12}$ |
| $M_r$ | 358,04 | 358.04 |
| Crystal system, space group | Triclinic, $P\bar{1}$ | Triclinic, $P\bar{1}$ |
| a, b, c (Å) | 5.20663(5), 7.32640(6), 9.24747(8) | 5.20542(4), 7.32708(5), 9.24798(6) |
| $\alpha, \beta, \gamma$ (°) | 108.9072(7), 105.4976(8), 92.3098(7) | 108.8728(6), 105.5716(6), 92.2943(6) |
| V(Å$^3$) | 318.456(5) | 318.380(4) |
| Z | 1 | 1 |
| *Data collection* | | |
| Temperature (K) | 100.00(11) | 100.0(3) |
| $\mu$ (mm$^{-1}$) | 1.250 | 1.250 |
| Absorption correction | Multi-scan [CrysAlisPro 1.171.42.84a (Rigaku Oxford Diffraction, 2023). Empirical absorption correction using spherical harmonics, implemented in SCALE3 ABSPACK scaling algorithm] | Multi-scan [CrysAlisPro 1.171.42.84a (Rigaku Oxford Diffraction, 2023). Empirical absorption correction using spherical harmonics, implemented in SCALE3 ABSPACK scaling algorithm] |
| $\theta_{min}$, $\theta_{max}$ | 2.4250, 79.0550 | 2.438, 79.501 |
| Number of measured and independent reflections | 174906, 14021 | 206434, 13955 |
| $R_{int}$ | 0.0363 | 0.0630 |
| $(\sin \theta/\lambda)_{max}$ (Å$^{-1}$) | 1.38 | 1.38 |
| *Spherical atom refinement* | | |
| R(F$^2$), wR(F$^2$), S | 0.0209, 0.0570, 1.058 | 0.0251, 0.0692, 1.073 |
| Number of reflections | 13896 [$I > 2\sigma(I)$] | 13187 [$I > 2\sigma(I)$] |
| Number of parameters | 125 | 125 |

| | | |
|---|---|---|
| $\Delta\rho_{max}$, $\Delta\rho_{min}$ (e Å$^{-3}$) | 0.90, -1.10 | 1.91, -1,29 |
| *Multipolar refinement* | | |
| R(F), wR(F$^2$), S | 0.0162, 0.0454, 1.4093 | 0.0191, 0.0475, 1.1565 |
| | [wR(F)=0.0229] | [wR(F)=0.0231] |
| Number of reflections | 13896 [I>σ(I)] | 13286 [F > 3σ(F)] |
| Number of parameters | 346 | 345 |
| $\Delta\rho_{max}$, $\Delta\rho_{min}$ (e Å$^{-3}$) | 0.68, -0.22 | 0.61, -0.65 |

**Table S2** Distance from the atoms to the bond critical points, electron density (ρ($r_{bcp}$)), Laplacian ($\nabla^2\rho(r_{bcp})$), principal curvatures, and ellipticity values at bond critical points from the evaluated models: MM1 (1st line), HAR1†† (2nd line).

| Bond | $d_1$†(Å) | $d_2$†(Å) | $\rho(r_{bcp})$( eÅ$^{-3}$) | $\nabla^2\rho(r_{bcp})$ ( eÅ$^{-5}$) | $\lambda_1$ | $\lambda_2$ | $\lambda_3$ | ε |
|---|---|---|---|---|---|---|---|---|
| Fe – O1 | 1.078 | 1.082 | 0.348 | 6.879 | -1.687 | -1.463 | 10.030 | 0.153 |
| | 1.047 | 1.114 | 0.382 | 5.905 | -1.880 | -1.821 | 9.606 | 0.032 |
| Fe – O5 | 1.044 | 1.041 | 0.408 | 8.481 | -2.085 | -1.574 | 12.140 | 0.325 |
| | 1.018 | 1.067 | 0.418 | 8.404 | -2.152 | -1.394 | 11.951 | 0.544 |
| Fe – O6 | 1.068 | 1.081 | 0.342 | 7.065 | -1.486 | -1.420 | 9.971 | 0.047 |
| | 1.043 | 1.104 | 0.371 | 6.938 | -1.835 | -1.295 | 10.068 | 0.417 |
| O1 – C1 | 0.852 | 0.404 | 2.618 | -11.580 | -27.361 | -25.747 | 41.528 | 0.063 |
| | 0.828 | 0.428 | 2.562 | -12.558 | -23.832 | -22.288 | 33.562 | 0.069 |
| O2 – C1 | 0.857 | 0.412 | 2.518 | -16.131 | -24.845 | -24.031 | 32.744 | 0.034 |
| | 0.835 | 0.434 | 2.503 | -13.846 | -22.883 | -21.296 | 30.333 | 0.074 |
| O3 – C4 | 0.879 | 0.419 | 2.314 | -14.495 | -20.971 | -20.660 | 27.136 | 0.015 |
| | 0.853 | 0.443 | 2.342 | -14.195 | -20.767 | -19.289 | 25.860 | 0.077 |
| O4 – C4 | 0.834 | 0.400 | 2.664 | -6.292 | -27.393 | -26.280 | 47.381 | 0.042 |
| | 0.810 | 0.423 | 2.738 | -12.524 | -26.077 | -22.895 | 36.448 | 0.139 |
| C1 – C2 | 0.757 | 0.739 | 1.840 | -16.995 | -13.114 | -11.367 | 7.486 | 0.154 |
| | 0.768 | 0.727 | 1.817 | -18.103 | -13.874 | -12.526 | 8.298 | 0.108 |
| C3 – C4 | 0.742 | 0.755 | 1.875 | -16.531 | -12.999 | -11.400 | 7.868 | 0.140 |

| | | | | | | | | |
|---|---|---|---|---|---|---|---|---|
| | 0.763 | 0.734 | 1.812 | -18.119 | -13.862 | -12.753 | 8.496 | 0.087 |
| C2 – C3 | 0.659 | 0.688 | 2.370 | -23.591 | -16.622 | -13.708 | 6.739 | 0.213 |
| | 0.677 | 0.669 | 2.336 | -28.713 | -18.703 | -14.500 | 4.490 | 0.290 |
| C2 – H2 | 0.730 | 0.340 | 1.851 | -18.452 | -17.087 | -16.920 | 15.554 | 0.010 |
| | 0.699 | 0.385 | 1.971 | -27.709 | -19.631 | -19.282 | 11.204 | 0.018 |
| C3 – H3 | 0.735 | 0.345 | 1.791 | -17.547 | -16.549 | -15.845 | 14.847 | 0.044 |
| | 0.723 | 0.384 | 1.890 | -25.776 | -18.738 | -18.610 | 11.572 | 0.007 |
| O5 – H5A | 0.772 | 0.182 | 2.249 | -53.116 | -41.591 | -40.673 | 29.148 | 0.023 |
| | 0.770 | 0.165 | 2.653 | -73.996 | -53.988 | -53.039 | 33.030 | 0.018 |
| O5 – H5B | 0.756 | 0.181 | 2.392 | -58.575 | -45.277 | -44.523 | 31.226 | 0.017 |
| | 0.773 | 0.177 | 2.544 | -68.317 | -48.011 | -47.081 | 26.775 | 0.002 |
| O6 – H6A | 0.743 | 0.186 | 2.404 | -49.130 | -42.284 | -41.489 | 34.643 | 0.019 |
| | 0.774 | 0.780 | 2.476 | -66.011 | -45.929 | -45.137 | 25.056 | 0.017 |
| O6 – H6B | 0.759 | 0.185 | 2.333 | -52.786 | -43.081 | -42.222 | 32.517 | 0.020 |
| | 0.778 | 0.180 | 2.511 | -66.011 | -46.629 | -45.838 | 25.566 | 0.011 |
| O3 – H3A | 0.849 | 0.234 | 1.519 | -20.228 | -24.034 | -23.114 | 26.921 | 0.040 |
| | 0.886 | 0.250 | 1.480 | -22.732 | -20.301 | -20.077 | 17.646 | 0.011 |
| O2 … H3A | 0.945 | 0.392 | 1.002 | -5.916 | -10.462 | -10.058 | 14.604 | 0.040 |
| | 0.950 | 0.327 | 0.937 | -2.243 | -9.957 | -9.853 | 17.567 | 0.011 |
| O2 … H5A | 1.415 | 1.074 | 0.070 | 1.256 | -0.242 | -0.168 | 1.666 | 0.438 |
| | 1.442 | 0.989 | 0.076 | 1.095 | -0.253 | -0.169 | 1.516 | 0.502 |
| O3 … H5A$^{i}$ | 1.283 | 0.706 | 0.137 | 1.882 | -0.731 | -0.594 | 3.207 | 0.231 |
| O4 ... H5B$^{ii}$ | 1.169 | 0.661 | 0.264 | 1.393 | -1.626 | -1.600 | 4.619 | 0.016 |
| O4 … H6A$^{iii}$ | 1.223 | 0.697 | 0.183 | 2.220 | -0.947 | -0.934 | 4.101 | 0.015 |
| O1 … H6B$^{iii}$ | 1.188 | 0.685 | 0.270 | 1.341 | -1.652 | -1.614 | 4.607 | 0.024 |

Symmetry codes: i: 1-x, 1-y, 1-z; ii: x, y-1, z-1; iii: x, y, z-1; iv: 1+x, y, z. $^{\dagger}d_1$ = distance from atom 1 to the bcp and $d_2$ = distance from atom 2 to the bcp. $^{\dagger\dagger}$: although Multwfn calculates the topological properties ignoring symmetry (for each pair of atoms, two values are calculated), only one value is shown for each pair of atoms in the HAR1 line because the values are numerically identical after rounding. There is no HAR1-corresponding line for the last four interactions because the procedure only computes interactions within a unit cell, not considering the crystal packing.

**Table S3** Values of $G(r_{bcp})$ (hartreeÅ$^{-3}$), $L(r_{bcp})$, $V(r_{bcp})$ (hartreeÅ$^{-3}$), $H(r_{bcp})$ (hartreeÅ$^{-3}$), $G(r_{bcp})/\rho(r_{bcp})$ and $|V(r_{bcp})|/G(r_{bcp})$ for Fe-O bonds and hydrogen bonds within the explored models. MM1 (1st line), HAR1†: (2nd line).

| Bond | $G(r_{bcp})$ | $L(r_{bcp})$ | $V(r_{bcp})$ | $H(r_{bcp})$ | $G(_{bcp})/\rho(r_{bcp})$ | $H(r_{bcp})/\rho(r_{bcp})$ | $\|V(r_{bcp})\|/G(r_{bcp})$ |
|---|---|---|---|---|---|---|---|
| Fe – O1 | 0.459 | -0.481 | -0.437 | 2.208E-02 | 1.319 | 0.063 | 0.952 |
| | 0.437 | -0.413 | -0.462 | -2.422E-02 | 1.143 | -0.063 | 1.055 |
| Fe – O5 | 0.576 | -0.593 | -0.558 | 1.745E-02 | 1.411 | 0.043 | 0.970 |
| | 0.580 | -0.588 | -0.572 | 8.183E-03 | 1.387 | 0.020 | 0.986 |
| Fe – O6 | 0.464 | -0.494 | -0.434 | 3.037E-02 | 1.356 | 0.089 | 0.935 |
| | 0.478 | -0.485 | -0.470 | 7.559E-03 | 1.286 | 0.020 | 0.984 |
| O2 … H3A | 0.530 | 0.414 | -1.474 | -9.441E-01 | 0.529 | -0.942 | 2.781 |
| | 0.616 | 0.157 | -1.389 | -7.731E-01 | 0.658 | 0.825 | 2.255 |
| O2 … H5A | 0.068 | -0.088 | -0.048 | 1.974E-02 | 0.973 | 0.282 | 0.710 |
| | 0.062 | -0.077 | -0.047 | 1.459E-02 | 0.816 | 0.192 | 0.765 |
| O3 … H5A$^{i}$ | 0.117 | -0.132 | -0.102 | 1.464E-02 | 0.854 | 0.107 | 0.875 |
| O4 … H5B$^{ii}$ | 0.152 | -0.097 | -0.207 | -5.481E-02 | 0.577 | -0.208 | 1.360 |
| O4 … H6A$^{iii}$ | 0.151 | -0.155 | -0.147 | 4.377E-03 | 0.825 | 0.024 | 0.971 |
| O1···H6B$^{iii}$ | 0.153 | -0.094 | -0.213 | -5.935E-02 | 0.567 | -0.220 | 1.387 |

Symmetry codes: i: 1-x, 1-y, 1-z; ii: x, y-1, z-1; iii: x, y, z-1; iv: 1+x, y, z. †: Although Multwfn calculates the topological properties ignoring symmetry, only one value is shown for each pair of atoms in the HAR1 line because the values are numerically identical after rounding. There is no HAR1-corresponding line for the last four interactions because the procedure only computes interactions within a unit cell, not considering the crystal packing.

**Table S4** Statistical parameters by resolution range. Exp_slow (1st line), Exp_fast (2nd line).

| Resolution (Å) | % complete | Average redundancy | Mean F² | Mean F²/sig(F²) | $R_{int}$ |
|---|---|---|---|---|---|
| inf-0.78 | 100.0 | 8.1 | 6196.40 | 63.22 | 0.023 |
| | 100.0 | 14.6 | 5497.30 | 45.31 | 0.043 |
| 0.78-0.62 | 100.0 | 10.3 | 1760.32 | 51.52 | 0.036 |
| | 100.0 | 19.7 | 1531.65 | 38.79 | 0.065 |
| 0.62-0.54 | 100.0 | 20.0 | 1034.21 | 63.92 | 0.040 |
| | 100.0 | 24.2 | 911.46 | 34.52 | 0.075 |
| 0.54-0.49 | 100.0 | 19.2 | 719.23 | 55.47 | 0.047 |
| | 100.0 | 21.9 | 620.13 | 28.40 | 0.093 |
| 0.49-0.46 | 100.0 | 16.8 | 492.23 | 48.80 | 0.053 |
| | 100.0 | 19.1 | 432.05 | 24.29 | 0.108 |
| 0.46-0.43 | 100.0 | 14.7 | 352.30 | 43.53 | 0.060 |
| | 100.0 | 16.3 | 315.91 | 21.54 | 0.118 |

| | | | | | |
|---|---|---|---|---|---|
| 0.43-0.41 | 100.0 | 12.7 | 268.78 | 41.48 | 0.062 |
| | 100.2 | 12.2 | 238.12 | 20.68 | 0.110 |
| 0.41-0.39 | 100.0 | 10.6 | 215.61 | 40.52 | 0.064 |
| | 100.0 | 9.2 | 201.67 | 21.71 | 0.090 |
| 0.39-0.38 | 99.3 | 8.2 | 154.69 | 35.03 | 0.069 |
| | 99.9 | 7.2 | 144.02 | 19.01 | 0.096 |
| 0.38-0.36 | 91.9 | 4.3 | 105.38 | 25.80 | 0.066 |
| | 99.2 | 3.8 | 100.07 | 14.29 | 0.091 |
| Inf-0.36 | 99.1 | 12.5 | 990.38 | 49.65 | 0.036 |
| | 89.1 | 14.8 | 1117.84 | 29.29 | 0.063 |

**Table S5** Atomic charges and volumes calculated by QTAIM for MM2 model.

| Atom | $q_\Omega$ (*e*) | $V_\Omega$ (Å$^3$) | Atom | $q_\Omega$ (*e*) | $V_\Omega$ (Å$^3$) |
|---|---|---|---|---|---|
| Fe(1) | 1.12 | 10.07 | H(3A) | 0.70 | 0.87 |
| O(1) | -1.26 | 15.18 | $Total_{Hmal}$ | -0.76 | 112.73 |
| O(2) | -1.27 | 17.07 | O(5) | -1.30 | 16.53 |
| O(3) | -1.18 | 16.06 | H(5A) | 0.76 | 1.27 |
| O(4) | -1.28 | 16.96 | H(5B) | 0.66 | 1.56 |
| C(1) | 1.60 | 5.41 | $Total_{w5}$ | 0.12 | 19.37 |
| C(2) | 0.05 | 11.30 | O(6) | -1.22 | 18.47 |
| C(3) | -0.24 | 12.41 | H(6A) | 0.64 | 1.66 |
| C(4) | 1.65 | 6.06 | H(6B) | 0.67 | 1.49 |
| H(2) | 0.10 | 7.61 | $Total_{w6}$ | 0.08 | 21.62 |
| H(3) | 0.37 | 3.79 | $Total_{FeHmal}$ | 0.00 | 317.49 |

**Table S6** Atom symmetries chosen for MM1, MM1_cutoff, and MM2 models.

| Atom | Symmetry | Atom | Symmetry | Atom | Symmetry | Atom | Symmetry |
|---|---|---|---|---|---|---|---|
| Fe1 | -1 | O5 | 1 | C4 | m | H5A | cyl |
| O1 | 1 | O6 | m | H2 | cyl | H5B | cyl |
| O2 | 1 | C1 | m | H3 | cyl | H6A | cyl |
| O3 | 1 | C2 | m | H3A | m | H6B | cyl |
| O4 | 1 | C3 | m | | | | |

**Table S7** Geometric parameters (Å, °) for MM1 (1st line), HAR1 (2nd line).

| | | | |
|---|---|---|---|
| | Bonds (Å) | | |
| Fe – O1 | 2.16014(13) | C2 – C3 | 1.3463(2) |
| | 2.15996(13) | | 1.3459(2) |
| Fe – O5 | 2.08553(14) | C3 – C4 | 1.4967(2) |
| | 2.08529(14) | | 1.4969(2) |
| Fe – O6 | 2.14705(14) | O3– C4 | 1.2965(2) |
| | 2.14695(14) | | 1.2958(2) |
| C1 – O1 | 1.25651(18) | O4 – C4 | 1.2338(2) |
| | 1.25663(19) | | 1.2337(2) |
| C1 – O2 | 1.2692(2) | C2 – H2 | 1.067(10) |
| | 1.2686(2) | | 1.084(8) |
| C1 – C2 | 1.4954(2) | C3 – H3 | 1.081(11) |
| | 1.4955(2) | | 1.107(9) |
| | Angles(°) | | |
| O1 – Fe – O5 | 97.326(6) | C1 – C2 – C3 | 129.642(14) |
| | 97.304(6) | | 129.648(15) |
| O1 – Fe – O6 | 93.179(6) | C2 – C3 – C4 | 130.216(14) |
| | 93.179(6) | | 130.228(16) |
| O5 – Fe – O6 | 92.588(6) | C3 – C4 – O3 | 120.218(13) |
| | 92.593(6) | | 120.191(14) |
| H5A – O5 – H5B | 106.4(10) | O1 – C1 – O2 | 122.782(15) |
| | 107.2(10) | | 122.781(15) |
| H6A – O6 – H6B | 104.4(10) | O4 – C4 – O3 | 121.791(17) |
| | 108.1(10) | | 121.819(17) |

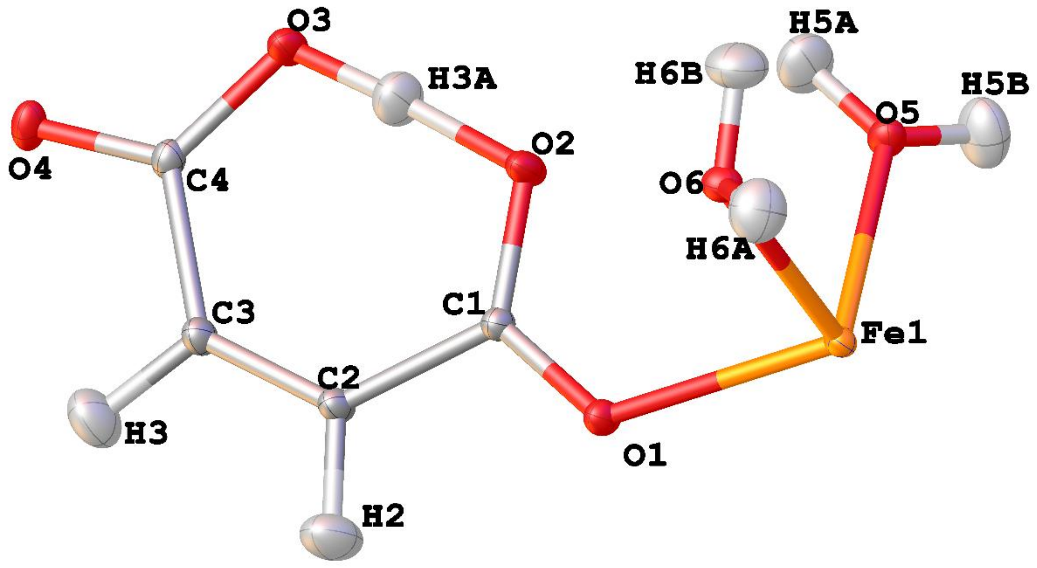


**Figure S1** FeHmal crystal structures generated by MM1_cutoff. Anisotropic displacement parameters are drawn at 50% probability.

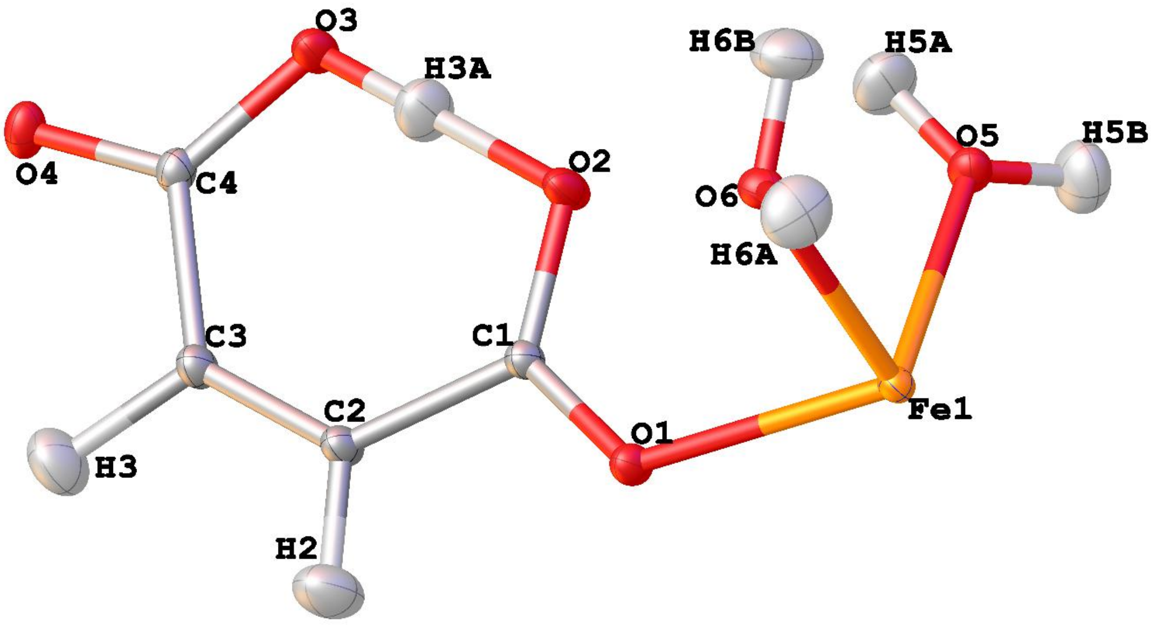


**Figure S2** FeHmal crystal structures generated by MM2. Anisotropic displacement parameters are drawn at 50% probability.

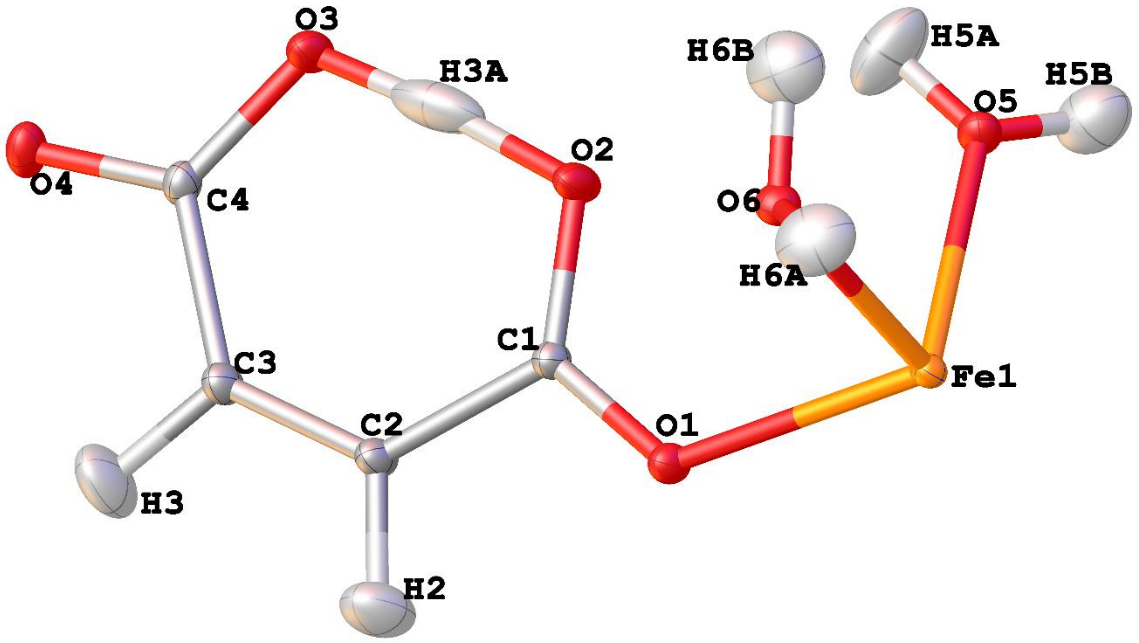


**Figure S3** FeHmal crystal structures generated by HAR1_cutoff. Anisotropic displacement parameters are drawn at 50% probability.

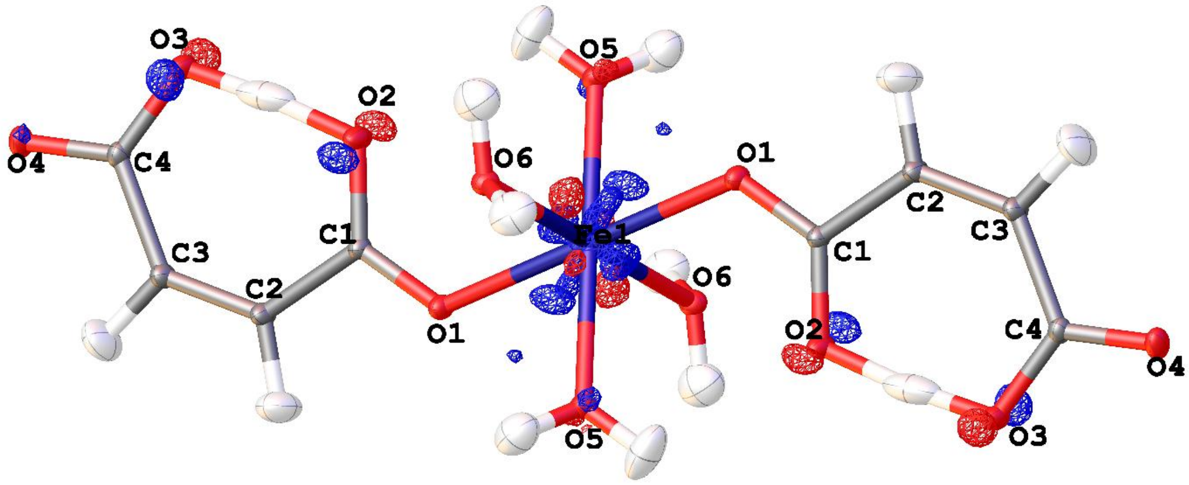


**Figure S4** Residual density map after HAR1_cutoff. Blue contours are positive and red contours are negative. Contour level 0.2 e Å$^{-3}$.

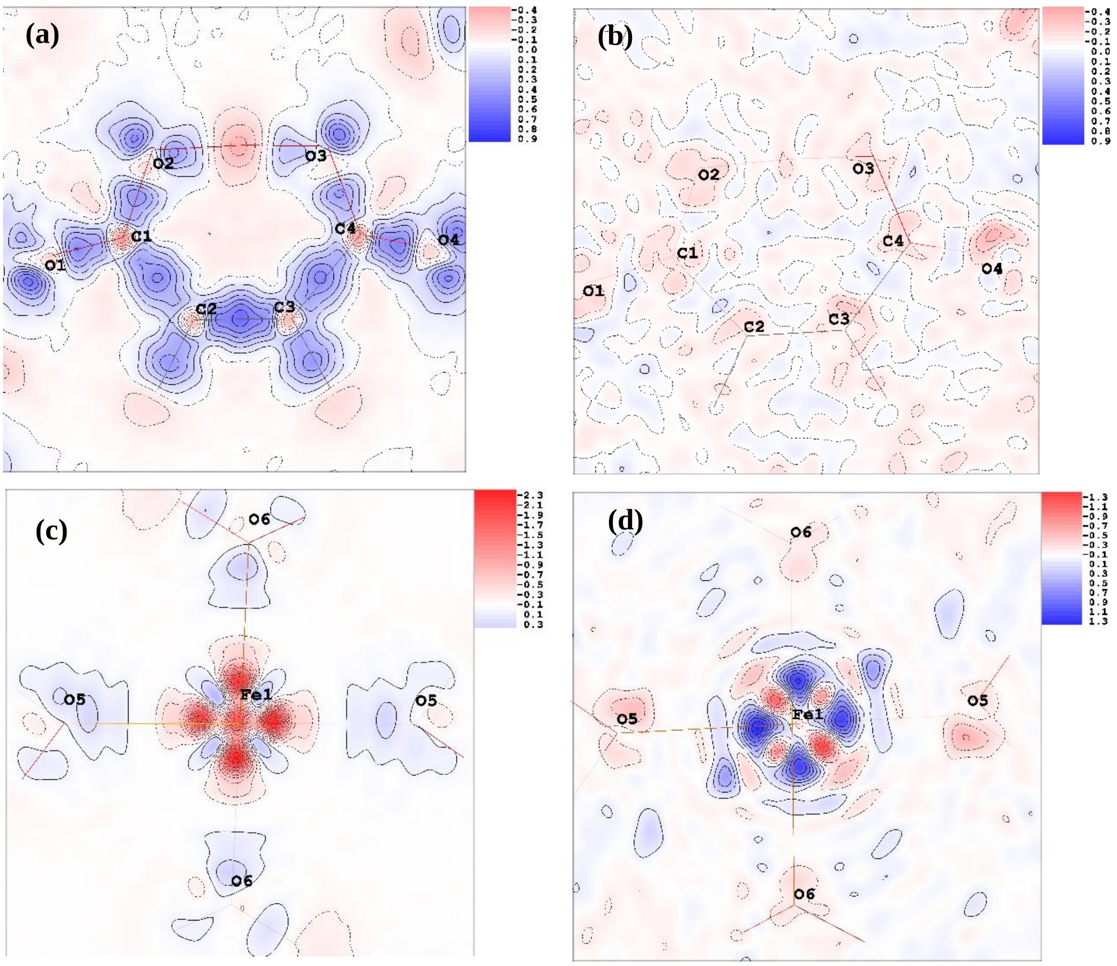


**Figure S5** Deformation and residual maps for the Hirshfeld atom refinement with the multiplicity equal to 1 for the iron atom. (a) Deformation map in the hydrogen maleate plane. (b) Residual map in the hydrogen maleate plane. (c) Deformation map in Fe1-O5-O6 plane. (d) Residual map in the Fe1-O5-O6 plane. Contours levels are given in e Å$^{-3}$, with positive contours in blue and negative contours in red.
